# Cryogenic light microscopy of vitrified samples with angstrom precision

**DOI:** 10.1073/pnas.2513583122

**Published:** 2025-12-03

**Authors:** Hisham Mazal, Franz-Ferdinand Wieser, Daniel Bollschweiler, Vahid Sandoghdar

**Affiliations:** ^a^Max Planck Institute for the Science of Light, Erlangen 91058, Germany; ^b^Max-Planck-Zentrum für Physik und Medizin, Erlangen 91058, Germany; ^c^Department of Physics, Friedrich-Alexander University of Erlangen-Nürnberg, Erlangen 91058, Germany; ^d^Max Planck Institute of Biochemistry, Planegg 82152, Germany

**Keywords:** cryogenic light microscopy, angstrom optical resolution, protein structure and assembly, correlative imaging, membrane proteins

## Abstract

High-resolution studies in structural biology are often limited by the challenges of crystallization and low contrast in the cellular native environment. The exquisite labeling specificity of fluorescence microscopy gets around these issues and allows superresolution microscopy, but to date, these works have used chemically fixed samples. To establish light microscopy as a workhorse in structural biology, two main requirements must be fulfilled: near-native sample preservation and near-atomic optical resolution. Here, we introduce single-particle cryogenic light microscopy (spCryo-LM) as a technique that satisfies these key criteria. We adapt established protocols from cryogenic electron microscopy (Cryo-EM) for shock-freezing samples and use a high-vacuum cryogenic shuttle system to transfer them in and out of a liquid-helium cryostat that houses a superresolution fluorescence microscope. By exploiting the enhanced photophysics at low temperature, angstrom precision can be achieved in localizing several fluorophores attached to proteins separated by a few hundred nanometers. We present various characterization studies on vitreous ice, single-molecule photoblinking behavior, and the effects of laser intensity and benchmark our method by resolving the heptameric membrane protein alpha-hemolysin in a synthetic lipid membrane. Additionally, we report on the technique’s capability to resolve membrane proteins in their native cellular membrane environment. spCryo-LM enables structural studies of proteins in their native environment without chemical fixation or protein isolation, and can be integrated with other superresolution or spectroscopic techniques. We believe our approach establishes light microscopy as a powerful tool in structural biology and sets the stage for correlative microscopy with Cryo-EM and related techniques.

## Significance

Despite the recent advances in structural biology, studies of proteins in their native environment face many challenges because cellular constituents generate a large background. As a result, molecules of interest are usually isolated or prepared in a synthetic form. Fluorescence labeling offers exquisite specificity, but superresolution studies have so far been performed on chemically fixed cells and have lacked sufficient resolution. We now introduce a method that preserves samples in their native state and achieves angstrom-level precision in localizing several fluorophores on single proteins spaced by a few hundred nanometers in fluorescence microscopy. This approach promises insights into the structural biology of proteins and biomolecular complexes, in particular when combined with cryogenic electron microscopy (Cryo-EM) in a correlative fashion.

Intensive efforts have been dedicated to the investigations of the structure and function of proteins and protein complexes (1–4), but their intricate details in the cell remain difficult to access. Advances in sample preparation (5) and cutting-edge hardware have sparked a revolution in cryoelectron microscopy (Cryo-EM), reaching atomic resolution in isolated protein macromolecules (6–9). However, a comprehensive insight into protein function can only be achieved in the native ultrastructural cellular context to ensure proper consideration of all biochemical and physical interactions. This ambitious goal is currently being pursued by cryogenic electron tomography (Cryo-ET) as a variant of Cryo-EM (10, 11), where protein structures and conformations are studied within cells and tissues preserved in their near-native states (12–15). A major drawback of Cryo-EM methods is that they necessitate a low-dose electron beam to avoid radiation damage, resulting in low signal-to-noise ratios (SNR). Thousands of individual particle images are usually averaged and classified in order to reach atomic resolution (11, 16). Consequently, the technique still faces significant challenges in identifying target molecules in their native state (11, 17, 18). For example, quantitative knowledge of the structure of integral membrane proteins (19–21), which account for 20 to 30% of the total proteome (22, 23), and their organization within the cell membrane remains scarce (24–26).

Fluorescence microscopy provides molecular specificity and exceptional sensitivity down to the single-molecule level, making it a workhorse for studying cellular and subcellular structures. The remarkable recent progress in superresolution microscopy has pushed the three-dimensional (3D) spatial resolution to 15 to 20 nm in routine studies (27), and state-of-the-art techniques such as minimal photon flux (MINFLUX) (28) and DNA-PAINT (29) have even reported precision on the subnanometer scale. Because this level of spatial resolution requires exceptional mechanical stability to counter unwanted effects caused by thermal molecular jitter, instrumental vibrations, and drifts, samples have been chemically fixed for room temperature (RT) investigations. The fact that chemical fixation can introduce artifacts and distort the system under study, however, makes this approach suboptimal (30–34).

To date, shock-freezing is considered to be the method that best preserves cellular and molecular structures. This cryogenic form of fixation provides near-native state preservation of biological samples with minimal disruption (35, 36). Having been invented in the context of Cryo-EM, this technique has also been used for cryogenic fluorescence microscopy, but mainly to help identify target proteins prior to measurements in an electron microscope (37). The majority of such setups operate at the liquid nitrogen (LN) temperature and atmospheric pressure (38–40), suffering from mechanical and thermal instabilities as well as condensation and devitrification (38); see review articles for details (37, 41). Recently, a commercially available instrument (42) has successfully integrated light microscopy with focused ion beam (FIB) and scanning electron microscopy (SEM). This system, which operates under high vacuum and at LN temperature, employs light microscopy for guiding the FIB milling process of a target region within a sample. The central challenges in previous works are 1) laser illumination is restricted to low intensities in order to prevent sample devitrification (43); 2) photophysical properties of fluorophores at low temperatures remain poorly understood (39, 40, 44); 3) constrained conformational changes of fluorescent proteins under cryogenic conditions reduce the prospects of photoactivation for localization microscopy (37); 4) applicability of special chemicals that enhance photoblinking is less effective due to hindered diffusion at low temperatures. As a result, optical resolution has generally remained in the range of 10 to 100 nm. Another recent work pushed cryogenic light microscopy to a higher localization precision in vitrified samples prepared on thick sapphire discs at liquid helium temperature (LHe) (32). However, this choice of substrate is not favorable for direct correlative Cryo-EM studies.

In this work, we report on an approach for resolving protein conformation and assembly with angstrom precision in their native state. By using commercially available transmission electron microscopy (TEM) grids as sample carrier, we devised a method that is readily compatible with Cryo-EM and correlative imaging. To achieve this, we engineered a high-vacuum cryogenic shuttle system that allows us to transfer shock-frozen vitrified samples in and out of a cryogenic optical microscope with minimal condensation and devitrification. We benchmark our method on a model system consisting of the heptameric membrane protein, alpha-hemolysin (αHL). The proteins are reconstructed in small unilamellar vesicles (SUVs), which are in turn used to create a synthetic lipid bilayer (SLB). We also report on the ability of our pipeline to interrogate transmembrane proteins in their native cellular membrane. The work flow developed here can be further used for investigations of other cell biological samples with a variety of optical techniques such as Raman spectroscopy (45).

## Results

### Cryogenic Light Microscopy of Vitrified Samples.

Previously, we introduced cryogenic localization microscopy in three dimensions (COLD), in which angstrom resolution was achieved by exploiting the stochastic photoblinking of single fluorescent labels on isolated protein molecules embedded in a polymer (46, 47). In the current work, we modify the experimental process to perform measurements on biological samples that are preserved in their near-native state via shock-freezing. Given that the method allows one to visualize the organization of single proteins, we also refer to it as single-particle cryogenic light microscopy (spCryo-LM) in analogy to single-particle cryogenic electron microscopy (spCryo-EM).

One of the common techniques for vitrification entails rapid immersion of a TEM grid, which supports a sample covered by a thin layer of water, into a cryogenic liquid (5). This process, called plunge freezing, achieves sufficiently high cooling rates for a sample thickness up to about 1 µm (48) and enables the preservation of macromolecules, protein crystals, viruses, cells, and even thin bacteria in their near-native states (49, 50). Subsequent to plunge freezing, the vitrified sample must be maintained below the critical temperature of 130 K to prevent ice crystallization (48). Additionally, exposure to air must be minimized to reduce contamination arising from condensation on the cold surface. To fulfill these conditions, we adapted a design strategy from Cryo-EM applications for use in our setup (51), which allows us to transfer vitrified specimens at high vacuum and LN temperature between different apparatuses.

**Fig. 1.**
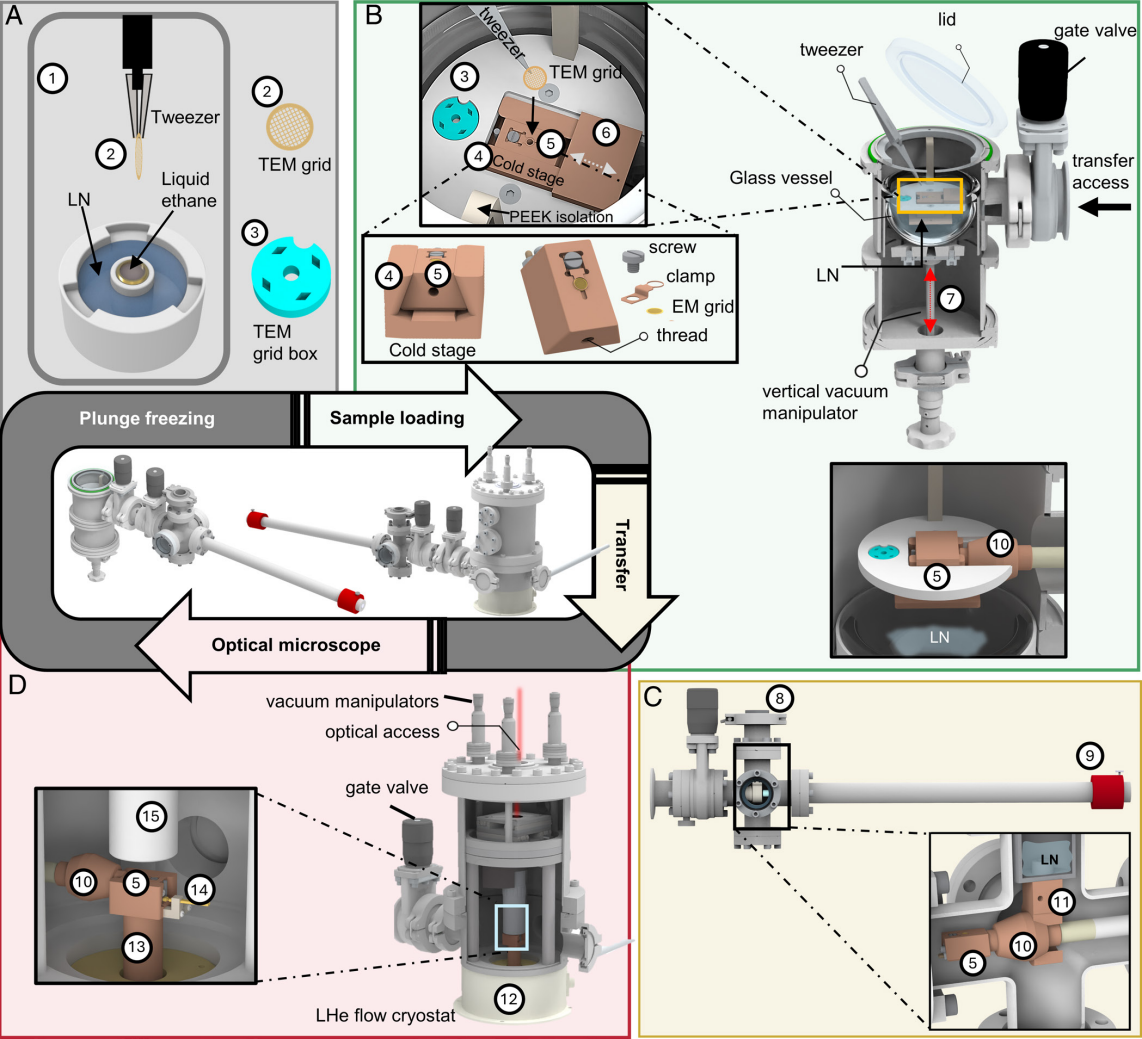
Cryogenic sample preparation and transfer. (*A*) Plunge freezing is achieved in a dedicated commercial instrument containing a cryogenic liquid (1). The sample on a TEM grid (2) is then placed and stored in a TEM grid box filled with LN (3). (*B*) A sample loading chamber uses a glass vessel to surround a cold stage (4) that holds a sample cartridge (5) with LN. The vessel can be lowered by a vacuum-tight manipulator (7). A lid can close the chamber for application of vacuum. A gate valve allows access from the side for the sample transfer. After the grid box (3) is transferred, the TEM grid is loaded and clamped onto the sample cartridge (see *Left* *Insets*). A sliding plate acts as an anticontaminator cover (6). (*C*) A vacuum-tight transfer shuttle can be flanged to the loading chamber and the cryostat via a gate valve. The essential components include a small LN dewar (8) and a vacuum-compatible manipulator (9). *Bottom* *Right* *Inset* of panel (*B*) shows the connection to the preparation chamber where the transfer shuttle is loading the sample cartridge after lowering the LN vessel. Inset of panel (*C*) shows a cross-section of the LN dewar. The manipulator rod fits through a c-shaped cold stage (11), and a conical taper receives the head (10), providing a perfect fit for efficient thermal conduction to the sample cartridge (5). (*D*) The transfer shuttle is connected to the cryostat which houses the optical microscope (12). *Inset* shows the connection of the cartridge carried by the transfer shuttle with a dedicated cold finger (13) in the cryostat. The microscope is based on a Janis-500 cryostat (12) with a series of modifications to accommodate a 3D piezo scanner and high-NA microscope objective (100×, 0.9 NA, 2 mm working distance) in vacuum (15, *Inset*) (47). The cold finger (13) was fitted with a dove tail to receive the sample cartridge, as well as a temperature sensor reader (14).

Fig. 1 sketches the principal workflow of shock-freezing a sample and its transfer from a preparation chamber into the cryogenic optical microscope. We first plunge freeze the sample in liquid ethane and then store it in a dedicated TEM grid box (Fig. 1*A*, positions 1-3). Next, the grid box is docked onto a sample-handling platform in an insulated glass vessel filled with LN inside a preparation chamber (Fig. 1*B*, positions 3-6). The platform is held by two polyether ether ketone (PEEK) isolation brackets connected to the top flange (Fig. 1*B* and *SI Appendix*, Fig. S1). The glass vessel is attached to a vacuum-tight vertical manipulator (Fig. 1*B*, position 7) that allows us to move it up and down during the transfer process. Fig. 1 *B*, *Top* *Left* *Inset* shows a close-up view of the sample-handling platform. A groove helps hold the TEM grid box (position 3), and a cold stage with a dovetailed structure (position 4) supports a sample cartridge (position 5, Fig. 1 *B*, *Bottom* *Left* *Inset*). An anticontaminator plate (position 6) can be slid over the sample to protect it during transfer (Fig. 1 *B*, *Top* *Left* *Inset*). The TEM grid is picked up from the grid box in LN, is mounted onto the sample cartridge and secured with a thin-profile clamp with a small screw (Fig. 1 *B*, *Bottom* *Left* *Inset* and *SI Appendix*, Fig. S2). To ensure good contact between the sample cartridge and the cold stage, we use two pairs of repelling magnets, press-fitted to the bottom of both the cartridge and the cold stage (*SI Appendix*, Fig. S2). A sensor is attached to the complementary holder close to the sample cartridge to monitor the chamber temperature (*SI Appendix*, Fig. S2).

Upon loading the TEM-grid, we slide the anticontaminator plate (Fig. 1*B*, position 6) onto the top of the sample cartridge to avoid any condensations or contaminations. Next, we close the lid of the preparation chamber and lower the glass vessel containing LN to prepare the transfer of the cartridge to the side (Fig. 1 *B* and *C*). The chamber is then evacuated to 0.1 to 10 mbar, as some LN remains in the glass vessel and does not fully vaporize within the duration of the transfer process (*SI Appendix*, Figs. S1–S3, *Supporting Text* 1, and *Supporting Protocol* and Movie S1). To suppress water vapor in the preparation chamber, we purge the preparation chamber with dry nitrogen throughout the entire process up until the evacuation step, during which the sample cartridge remains fully immersed in liquid nitrogen. A manipulator from a high-vacuum shuttle (10^−6^ mbar) operating at LN temperature is used to transfer the cartridge from the loading chamber (Fig. 1*C*) to a cryostat that houses the optical microscope, precooled at LHe temperature (Fig. 1*D*). Fig. 1*C* shows the full transfer shuttle. The essential components include a small LN dewar (position 8) and a linear vacuum-compatible manipulator (position 9). The manipulator rod contains a large-mass conical head (position 10) made from oxygen-free copper, which holds a screw to load the sample cartridge through a threaded hole on its side (Fig. 1 *B*, *Bottom* *Left* *Inset*, Fig. 1 *C*, *Inset*, and *SI Appendix*, Fig. S1).

After loading the sample cartridge (position 5) from the preparation chamber into the transfer shuttle under low vacuum, the cartridge is brought to contact with the LN temperature cold stage (position 11), via the tool head. This enables the sample to maintain its temperature below the devitrification point (*SI Appendix*, Fig. S1). Next, the gate valve of the preparation chamber is closed to allow the transfer shuttle to reach high vacuum. Then, the gate valve of the transfer shuttle is closed, the transfer shuttle is disconnected from the preparation chamber, and it is connected to the cryostat through a KF50 flange (Fig. 1*D*). Then, we transfer the cartridge to the cold stage inside the optical microscope, which is kept under high vacuum and cooled to LHe temperature (Fig. 1*D*, position 12 to 15 and *SI Appendix*, Fig. S3). A comprehensive workflow, characterization, and description are provided in the *SI Appendix*, Figs. S1–S4, *Supporting Protocol*, and *Supporting Text* 1 and Movie S1. The procedure can also be executed in the reverse order for grid extraction and subsequent analysis and correlative imaging.

To obtain sufficiently large numbers of photons during each blinking on-time, it is desirable to excite a molecule at a high rate, requiring laser intensities in the range of 0.6 to 1 kW/cm^2^. This level of light intensity has been reported to cause severe sample devitrification at 77 K, especially in the case of TEM grids with a holey support film (39, 43). We, thus, screened several grid materials at 8 K. We found that holey support films are unstable, buckle, and tear at high laser intensities ~0.3 to 0.6 kW/cm^2^ (*SI Appendix*, Fig. S5 and Movie S2). As a result, we opted to work with UltrAufoil (UF) TEM grids R2/2 200 mesh, which have been shown to offer superior mechanical stability (52) and to handle high optical intensities (~0.6 kW/cm^2^) (43), particularly when using light in the red spectral range (53), thereby maintaining stable vitreous ice condition (*SI Appendix*, Fig. S5).

**Fig. 2.**
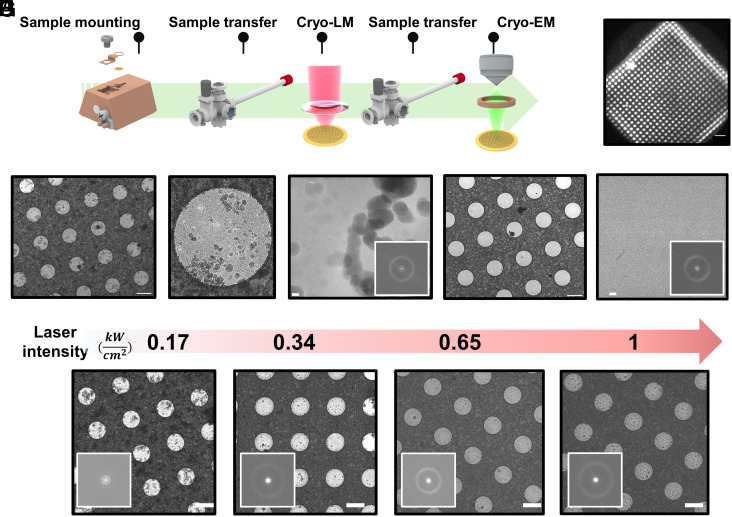
Sample transfer characterization and vitreous ice stability. (*A*) Schematics of the characterization procedure. (*B*) An example of the fluorescence image from a single square region on the TEM grid recorded in the cryostat. The bright regions depict holes containing frozen aqueous solution doped with nM fluorescent molecules. (Scale bar is 10 µm.) (*C*) Samples are retracted after the measurements in (*B*), in reverse order to that shown in Fig. 1 (see text). They are then taken to a cryoelectron microscope (Titan Krios G2, 300 kV, K3 EFTEM). Micrograph of the holey gold foil exposed to 0.65 kW/cm^2^ laser intensity imaged. The image shows minimal traces of transfer ice. (Scale bar is 2 µm.) (*D*) Close inspection of the holes from the image in panel (*C*) indicates sparsely faint contaminations. Hole diameter is 2 µm. (*E*) Close-up image of the hole in panel (*D*) shows intact vitreous ice covered with black contamination, reminiscent of atmospheric ice (small water vapor) (56). Power spectra analysis (*Inset*) of the same image indicates pure vitreous ice condition. (Scale bar is 20 nm.) (*F* and *G*) A holey gold foil TEM grid that was transferred and exposed to laser radiation as described in (*C*–*E*), but with pinhole disc cover (100 µm hole) on *Top*. The images show that the grid is very clean with minimal transfer ice and no water vapor contamination. Scale bar in (*F*) and (*G*) are 2 µm and 20 nm, respectively. Power spectra analysis of the same image (*Inset* of *G*) indicates pure vitreous ice condition. These data show that water vapor contamination can be avoided. (*H*) Vitreous ice stability as a function of laser intensity. TEM grid micrograph at intermediate magnification and the associated power spectra (*Inset*) of vitreous ice exposures in holes at high magnification (105 kx, equivalent to 0.85 Å/px). The data indicate no devitrification takes place with laser irradiation up to ~1 kW/cm^2^ (see additional measurements in *SI Appendix*, Figs. S6 and S7). (The scale bar is 2 μm.)

To investigate the quality of the entire vitrification procedure, including the sample transfer and the effect of laser illumination at our working wavelength of 645 nm, we examined the vitrified TEM grids using Cryo-EM (Fig. 2*A*). To validate proper blotting vitrification with a visual check in our optical microscope, we added ~20 nM iFlour647 dye molecules to pure aqueous samples (Fig. 2*B* and *SI Appendix*, *Supporting Text* 2). We tested the performance of our transfer system by transferring a UF TEM grid into the optical microscope, following the procedure described above and exposing it to 0.65 kW/cm^2^ in our cryostat at liquid helium temperature. The samples were subsequently retracted from the cryostat and were examined using Cryo-EM imaging (Fig. 2 *C*–*E*). Micrographs with a total electron dose of 60 e^−^/Å^2^ at an equivalent magnification of 0.85 Å/px were recorded. The calculated power spectra of the vitreous ice exposures in holes, i.e., the modulus of their Fourier transforms to the second power, was used as a quantitative measure for the presence of devitrified ice. The occurrence and intensity of crystalline ice diffraction rings are generally used to assess the presence of unwanted crystalline ice, with characteristic bands between 6 and 30 Å (54, 55).

As depicted in Fig. 2*C*, the micrograph indicates minimal transfer ice that occurred during the entire grid transfer procedure although one also finds some faint, sparse contamination covering the grid. A close inspection of the contamination on one of the holes (Fig. 2 *D* and *E*) reveals features reminiscent of atmospheric ice contamination, similar to what was observed in previous studies (53, 56). To confirm this, we first transferred a holey support film following the same procedure. As shown in *SI Appendix*, Fig. S6, this type of contamination covers the entire grid. We note that based on the power spectra analysis, the vitreous ice in such samples is completely intact and no devitrification was observed. Next, we transferred another UF grid, but this time included a 100 µm pinhole disc cover on top and exposed it to 0.65 kW/cm^2^ in our microscope cryostat. The grid was then retracted and examined using Cryo-EM. As shown in Fig. 2 *F* and *G*, the grid was very clean with minimal transfer ice and no water vapor contamination (see also *SI Appendix*, Fig. S6). This result lets us conclude that the source of this contamination was indeed surface contamination from water vapor condensing onto the sample cartridge during the transfer process. We attribute this to the low vacuum level of the preparation chamber during that step. Further engineering of the transfer process, such as integration of a mechanical shutter on top of the sample cartridge, will help reduce water vapor contamination.

To assess the laser effect on devitrification, we exposed different grids to varying laser intensities (0, 0.1, 0.3,0.6, and 1 kW/cm^2^) in the cryostat. Each TEM grid was illuminated for approximately 1 to 3 h in total (*SI Appendix*, *Supporting Text* 2). As depicted in Fig. 2*H*, we found no notable devitrification for laser irradiation up to 1 kW/cm^2^ (*SI Appendix*, Fig. S7, *Supporting Text* 2, and *SI Materials and Methods*). This outcome can be attributed to a combination of effects, such as the low base temperature in our setup (~8 K), the TEM grid material, and laser wavelength (53).

### Photophysics Characterization.

**Fig. 3.**
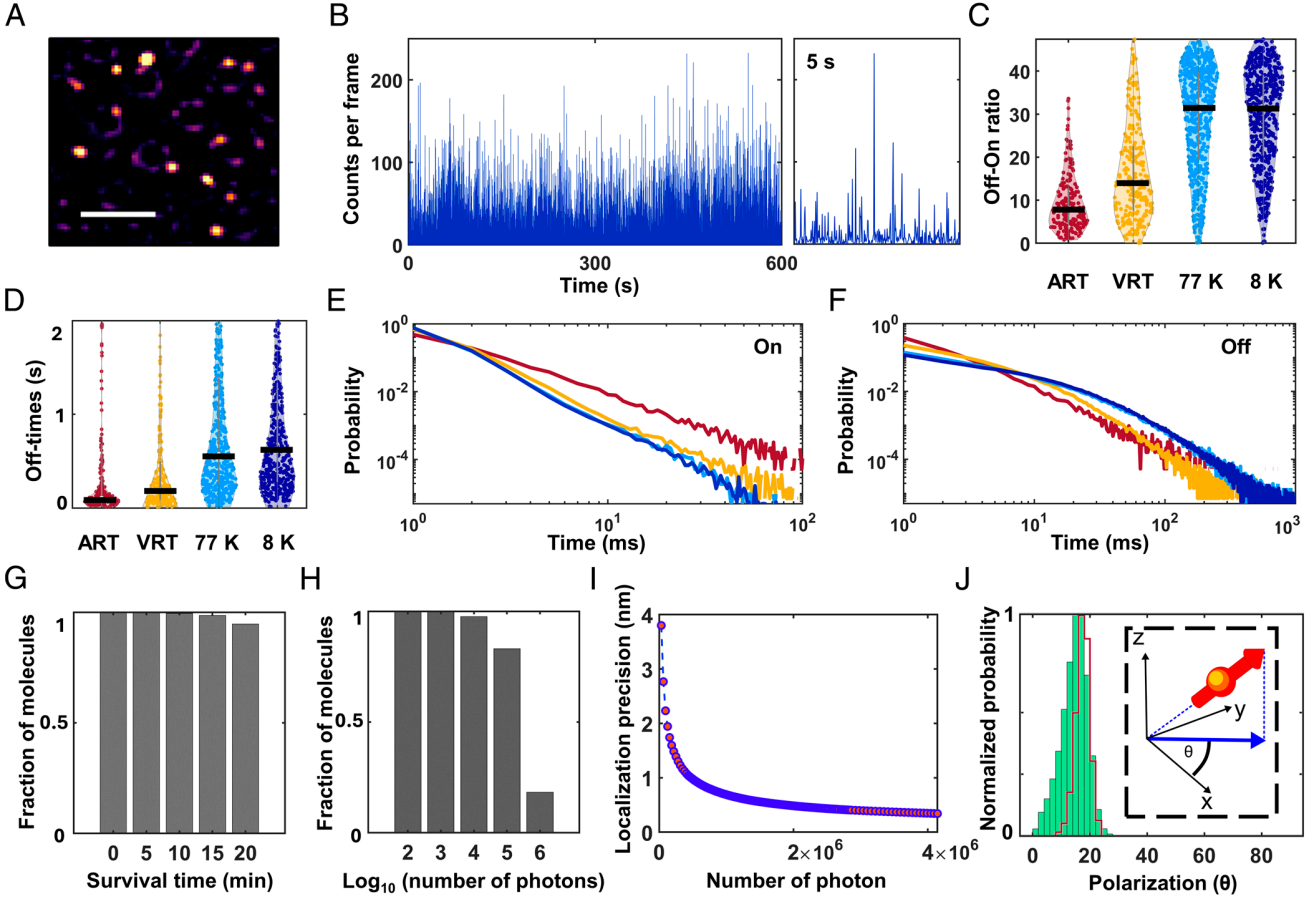
Photophysics characterization of fluorescent molecules. (*A*) Fluorescence image of a vitrified aqueous solution of ATTO647N recorded on a UF TEM grid. (Scale bar is 5 µm.) (*B*) Exemplary intensity time traces of a single dye molecule from panel (*A*) show large off-times and short on-times suitable for superresolution imaging. *Right* panel shows a close-up of a 5 s interval. (*C*–*F*) Photophysics of ATTO647N embedded in a PVA matrix (*SI Appendix*, *SI Materials and Methods*). (*C*) Off-on ratios obtained from a large number of molecules at different conditions. ART: ambient room temperature (N = 195); VRT: vacuum room temperature (N = 248); 77 K: vacuum, LN temperature (N = 558); 8 K: vacuum, LHe temperature (N = 536). The black horizontal lines indicate median values. (*D*) Distribution of off-times for each condition. Color code is the same as in panel (*C*). (*E* and *F*) Probability distributions of the on and off times presented on log–log plots. Color code is same to that in (*C* and *D*). (*E*) Fitting the on-times data with power law model yields power exponents of 2.1, 2.62, 2.90, and 2.95 for ART, VRT, 77 K, and 8 K, respectively. (*F*) We found that off-time distribution at ART can be best described by a power law behavior, with a power exponent of 1.93. On the other hand, off-time distributions at VRT, 77 K, and 8 K are more complex, following multiexponential behavior of more than 3 orders. (*G*–*I*) Survival time histogram shows that >90% of the molecules survived for at least 10 min, allowing the collection of >1 × 10^6^ photons and, thus, a localization precision down to the angstrom level. Localization precision better than 1 nm can be achieved if one uses >3,000 localizations events. (*J*) Polarization histogram of a single molecule indicating a stable and narrow distribution in vitreous ice, suitable for distinguishing individual molecules based on their orientations. The histogram becomes narrower (red line) when filtering the data to include only frames with a high photon count. *Inset* shows the projection of the dipole orientation onto the angular range [0° to 90°]. Panels *G*–*J* were obtained from vitrified ATTO647N, as in (*A*).

Having validated that our sample remains vitrified during laser illumination, we sought to characterize photoblinking in vitreous ice because the photophysics of dye molecules is known to be highly sensitive to their material environment (57). Here, we recorded the fluorescence from a vitrified solution of ATTO647N in water on UF TEM grids. As depicted in Fig. 3*A*, molecules were spread sparsely on the TEM grid. Fig. 3*B* shows that we obtain a photon count of ~5,000/s and observe fast spontaneous blinking with an average off-on time ratio of ~30, i.e., much longer off-times than on-times (Movies S3 and S4). This feature enables the resolution of multiple fluorophores below the diffraction limit (46, 58). Some previous studies reported weak blinking behavior at LN temperature, both under atmospheric pressure and in high vacuum (40, 59) while other work at high vacuum and LHe temperature (32) report strong photoblinking in line with what was observed in our work.

Next, we discuss the effect of vacuum and temperature. First, we imaged the ATTO647N sample at RT and ambient atmospheric pressure (ART). Then the same sample was imaged at RT but under high vacuum (VRT), followed by LN and high vacuum, and finally by LHe and high vacuum. To investigate the same sample under these conditions, we opted to record the data in hydrophilic polymer polyvinyl alcohol (PVA). We validated that the photophysical behavior was the same for both vitreous ice and the polymer (*SI Appendix*, Fig. S8). As depicted in Fig. 3*C*, we found that a moderate vacuum (~10^−4^ mbar) significantly enhances the off-on ratio as compared to atmospheric pressure by about a factor of two. Moreover, we observed that decreasing the temperature from RT to LN or further down to 8 K enhanced photoblinking by roughly another factor of two relative to ART. We analyzed the off-dwell and on-dwell times (*SI Appendix*, Fig. S9) by determining the median value from all the molecules and found that this enhancement results from both an increase in off-times and a decrease in on-times. As depicted in Fig. 3*D*, the median off-time increases as a function of pressure by a factor of 4, as a function of LN temperature by an additional factor of 3.2, and slightly improved by an additional factor of 1.1 at 8 K. This trend remains similar if one considers the median value of all on/off events from all molecules (*SI Appendix*, Fig. S9). We note that our estimate of the on-times is limited to our frame rate of ~14 ms. We also found similar effects in other far-red organic dyes such as Cy5, iFluor 647, and Alexa Fluor 647 (*SI Appendix*, Fig. S9). The on-time distribution (Fig. 3*E*) indicates a power-law behavior (60, 61) with an increasing power exponent as the temperature decreases. The off-time distribution (Fig. 3*F*) also follows a power-law behavior at ART (60, 61), but it shifts to a biexponential behavior as vacuum is applied and the temperature is reduced. This behavior is a consequence of the heterogeneity in the molecules’ local environment.

In addition to organic molecules, we also tested the photophysics of far-red fluorescent proteins, such as small Ultra Red Fluorescent Protein (smURFP) (62) and observed a similar behavior (*SI Appendix*, Fig. S9 and Movie S5). We emphasize that blinking is completely spontaneous, i.e., without the use of any special buffer conditions as required in techniques such as stochastic optical reconstruction microscopy (STORM) (63). In summary, while not all photoblinking effects at cryogenic temperatures are yet fully understood, our work shows that the combination of vacuum and low temperature provides favorable conditions for the use of spontaneous photoblinking in single-molecule localization microscopy at cryogenic temperature.

Another advantage of cryogenic temperatures, besides superior sample preservation and favorable photoblinking, stems from the fact that fluorophores remain stable against photobleaching for extended periods of time (Fig. 3*G*). This allows one to detect several orders of magnitude more photons from a single molecule than at ambient conditions (Fig. 3*H*). It is, indeed, this feature that is responsible for localization precisions in the range of a few angstroms (Fig. 3*I*).

### Superresolution Imaging in Vitrified Samples.

In order to resolve multiple fluorophores below the diffraction limit, one needs to isolate and localize each fluorophore separately. In our approach, we exploit the fixed dipole orientations of fluorescent molecules and their stochastic photoblinking at low temperature to separate them (46, 58). To do this, the fluorescence signal of a molecule is passed through a polarizing beam splitter, and its image is recorded on two cameras. A ratiometric analysis of the intensities of the resulting point-spread functions (PSF) allows us to project its transition dipole moment (see *Inset* of Fig. 3*J*) (58). In this case, the polarization angle (θ) is defined as $\theta =arctan\sqrt{\frac{I_{y}}{I_{x}}},$ for $\theta \epsilon [0,\frac{\pi }{2}]$. Fig. 3*J* plots an exemplary histogram of the polarization signal recorded from a single fluorophore.

It was previously shown that imaging fixed dipoles with a substantial axial component introduces considerable localization inaccuracies (64, 65). The consequences of this effect on our current work are negligible because 1) our wide-field illumination preferentially excites the in-plane components, and 2) the modest numerical aperture of 0.9 in our detection eliminates radiation beyond the critical angle and, thus, the majority of emission from the axial components of molecular dipoles. Hence, by discriminating against less bright fluorescence signals, we minimize the effect of dipole bias in localization accuracy (58); see *SI Appendix*, Fig. S10. We also note that several solutions have been recently proposed to significantly reduce this effect even for high-NA imaging (66, 67).

**Fig. 4.**
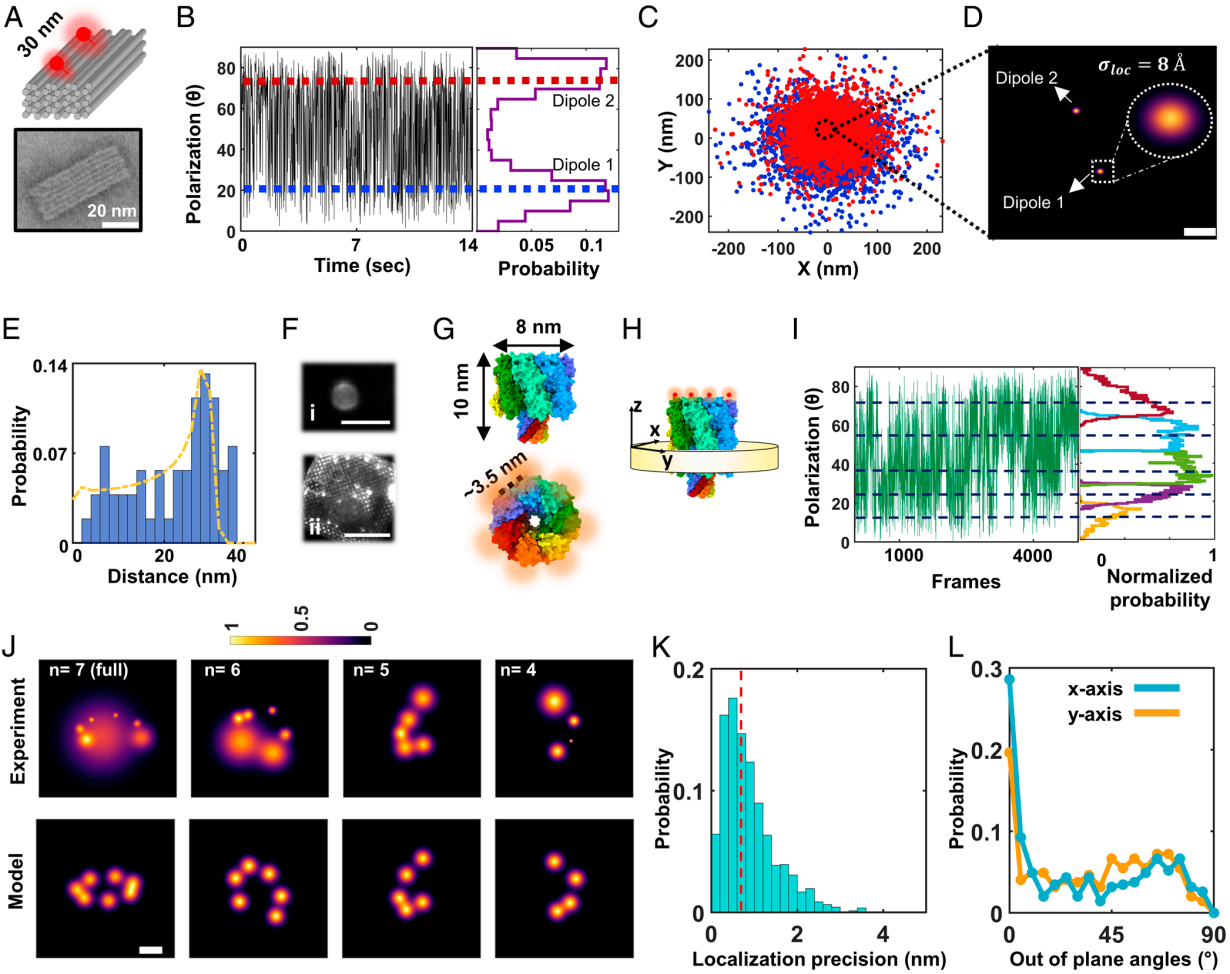
Superresolution imaging in vitreous ice with Ångstrom precision. (*A*) PF3 cuboid DNA origami structure with 2 ATTO647N molecules labeled at separation of 30 nm. *Top* panel is a schematic, and *Bottom* panel is a TEM image. (*B*) Polarization time trace obtained from one PSF of the DNA origami sample. The trace shows two peaks at orientations of ~20° and ~75° (blue and red lines). We note that this trace is not a continuous time trace, but it rather represents the on-states of the fluorophores during the recording, i.e., off-times were removed (see *SI Appendix*, Fig. S11 as an example). (*C*) Scatter plot of the localizations from the polarization events obtained from the two cameras. (*D*) By clustering the frames associated with each polarization state (using DISC algorithm, see *SI Appendix, Materials and Methods*), we generate a 2D resolved image which depicts the position of each fluorophore. The Gaussian width of the position reflects the localization uncertainty. (Scale bar is 10 nm.) (*E*) Distance histogram obtained from 53 projections with a fit (yellow line) using a model (*SI Appendix*, *SI Materials and Methods*). (*F*) Vitrified GUVs and SLB imaged in our cryogenic microscope. (i) is a GUV, and (ii) is SLB with ~100 nM fluorescence concentration. Scale bar is 5 μm and 50 μm respectively. (*G*) X-ray resolved structure of αHL (PDB: 7ahl), where each protomer is represented with a different color. The protein forms a symmetric heptamer, where the distance between the protomers is ~3.5 nm measured from the last amino acid at the C-terminal side. Red spots in the *Lower* panel indicate the positions of the fluorophores. (*H*) Axis definition of αHL for protein orientation discussion. (*I*) Exemplary polarization time trace from a single PSF shows 5 polarization states. The segmented histograms indicate the polarization states over time (see *SI Appendix, Materials and Methods*, and *SI Appendix*, Fig. S14). (*J*) 2D resolved images of single molecules resolved with different examples of 7, 6, 5, and 4 polarization states (*Top*). The projection of each was fitted to a model structure generated from PDB:7ahl (*Bottom*), revealing a very good agreement within our estimated distance error and localization precision. (Scale bar is 3 nm.) (*K*) Localization precision histogram from the recorded molecules (N = 345). (*L*) Probability of out-of-plane orientation of the particles in the range of [0° to 90°], as estimated from a simulated annealing algorithm (*SI Appendix*, *SI Materials and Methods*).

As a first demonstration, we imaged DNA origami nanorulers that contained two ATTO647N dye molecules separated by 30 nm (Fig. 4*A*). We loaded 3 μL of this sample onto a UF TEM grid, followed by plunge-freezing. The vitrified sample is transferred and imaged using the cryogenic optical microscope (Movie S6). While the fluorophores are free to rotate before shock-freezing, they become fixed in random orientations during this process. Fig. 4*B* showcases 2D projections of two polarization components at ~20° and 75°, and Fig. 4*C* displays their corresponding PSFs. By annotating the fluorophores along the time trace according to their polarization angles and clustering the spatial coordinates that we extract from the corresponding images, we measure the position of each fluorophore with angstrom precision (Fig. 4*D*, see also *SI Appendix*, Fig. S11). Fig. 4*E* depicts the distance histogram obtained from 53 different projections (N = 53). The histogram shows a main peak at ~30 nm with a long tail toward smaller distances, as expected for 2D sampling of different molecular orientations in 3D. Fitting the histogram with a model that considers this effect (*SI Appendix*, *SI Materials and Methods*) deduces a distance of 30.8 nm (Fig. 4*E*, yellow line). Additional analysis and data on another DNA origami can be found in *SI Appendix*, Fig. S12.

### Membrane Proteins Reconstructed in a Synthetic Lipid Membrane.

In this section, we benchmark our method by studying membrane proteins reconstructed in a synthetic lipid membrane. We demonstrate the ability of the technique to preserve and image model membranes on the TEM grid. Fig. 4*F* shows that giant unilamellar vesicles (GUVs) and supported lipid bilayer (SLB) could be preserved during the vitrification process and imaged via fluorescence labeling (*SI Appendix*, *SI Materials and Methods*). To reconstitute proteins into the synthetic lipid membranes, we chose αHL from *Staphylococcus aureus* (Fig. 4 *G* and *H*) as a model system, which is known to form a stable structure of seven protein subunits in the presence of a lipid environment (68, 69). We specifically labeled monomeric αHL via an engineered 6x histidine tag located at its C-terminal domain, using Tris-NTA iFlour 647 fluorophore (~ nM binding affinity, see *Materials and Methods* for further information). The protein was then reconstituted in 200 nm SUVs prepared form 1,2-diphytanoyl-sn-glycero-3-phosphocholine (DPHPC) and incubated with plasma-treated UF TEM grids with 2 nm carbon to form an SLB. The grid was plunge frozen and transferred to our cryogenic microscope (Movie S7). We elaborate on the validation of protein reconstitution in SUVs in *SI Appendix*, *SI Materials and Methods* section and *SI Appendix*, Fig. S13.

For each detected PSF in the sample, we calculated the polarization time trace from the ratio of the intensities imaged onto the two cameras, and selected particles that had three or more polarization components and maintained a maximum distance of 10 nm. While in some cases the polarizations might be very clearly resolved, distinguishing a large number of polarization states can be less trivial in other cases. To obtain a robust assessment, we employed an unsupervised clustering algorithm based on Bayesian information criteria (BIC) to determine the optimal number of polarization components (70). We validated this approach using segmentation analysis (*SI Appendix*, Figs. S13 and S14 and *SI Materials and Methods*). The *Left* panel of Fig. 4*I* shows an example of the time trace for five polarization components while the right panel marks the five components of the accumulated histogram, identified by the algorithm.

After identifying the individual fluorophores on an αHL particle, we localized each of their PSFs. In Fig. 4*J*, we present several cases of 2D-resolved images from particles that contained 4-7 labels. Fig. 4*K* plots the distribution of the localization precisions from individual molecules detected on the particles that were included in the analysis, yielding a median localization precision of 7 Å (N = 345 molecules). This enables us to resolve even the fully labeled heptameric configuration of αHL, where the smallest distance between protomers is around 3.5 nm (Fig. 4*J*, n = 7, *Upper* panel; see also *SI Appendix*, Fig. S15 for other 2D projections). We fitted the 2D-resolved image to a projection that was model generated based on the known crystal structure (PDB:7ahl) (69). The outcome (Fig. 4 *J*, *Lower*) indicates a good match between our data and the model within the estimated median distance error of 1.8 nm (*SI Appendix*, Fig. S15).

The 2D projection of the fluorophores on a membrane protein also allows us to assess the plane in which it is situated. The data in Fig. 4*L* reveal that, as expected, orientations out of the membrane plane are not favored. The distribution tail at large angles might indicate the presence of intact vesicles in the sample, where αHL can freely adopt any 3D random orientation. For example, the full heptamer configuration in Fig. 4*J* (n = 7) is rotated by 3° about the x-axis and 56° about the y-axis with respect to a fully flat (top view) structure illustrated in Fig. 4*H*. In another case, the projection with 6 fluorophores (n = 6) indicates rotation by 37° about the x-axis and 3° about the y-axis. As demonstrated in our earlier works, we can calculate the fluorophore configurations in 3D by taking advantage of the 2D projection of particles at different orientations and applying the same algorithm used in Cryo-EM (46). In particular, we derived the 3D reconstruction of the αHL by using particles that were labeled with seven fluorophores (*N* = 20). *SI Appendix*, Fig. S16 shows the outcome. We emphasize that in doing so, we neither made model assumptions, nor imposed any symmetry conditions (Fig. 4 and *SI Appendix*, Fig. S16). While providing a direct approach, the yield of this procedure is low because the labeling strategy in our work was based on affinity rather than a covalent bond. In other words, particles in which all domains are labeled are rare. Moreover, resolving a large number of fluorophores in one protein particle requires optimal blinking and polarization behavior from each fluorophore (*SI Appendix*, Figs. S15 and S17). Indeed, the overall high throughput of our imaging platform lets us reach a sufficient level of statistics despite stringent filtering criteria. However, it would also be interesting to explore alternative approaches. Below, we discuss a strategy for selecting underlabeled particles and analyzing the distribution of measured distances for cases, where a known symmetry can be assumed for the structure.

**Fig. 5.**
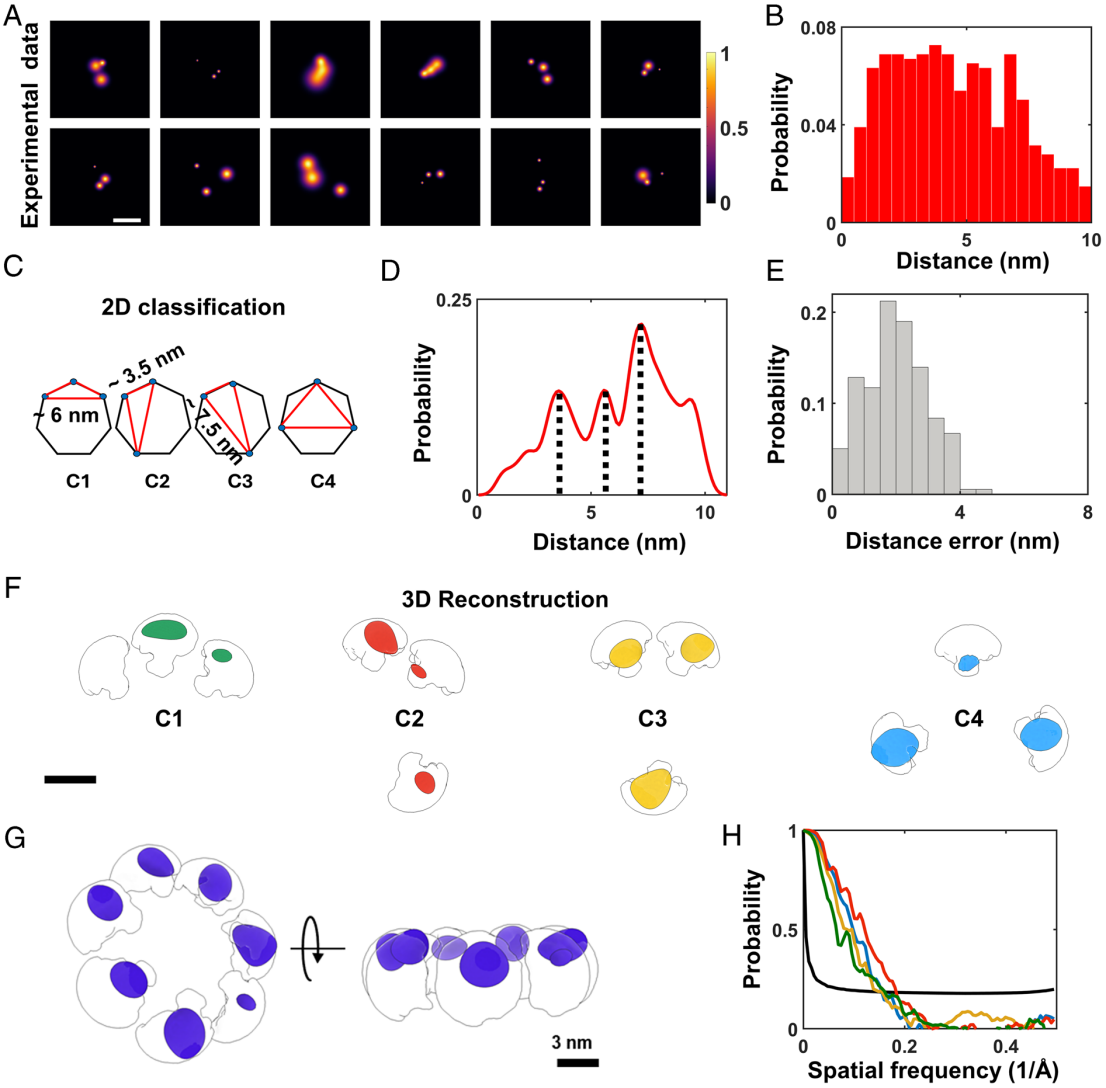
2D classification and 3D reconstruction of αHL. (*A*) Twelve examples of 2D images of single molecules resolved from PSFs with 3 polarization states (see also *SI Appendix*, Fig. S18). (Scale bar is 5 nm.) (*B*) Pair-wise distance histogram calculated from 179 2D projections such as those shown in (*A*). (*C*) Heptameric particles labeled with 3 fluorophores sustain triangles that fall into 4 categories, involving side lengths of ~3.5, 6, and 7.5 nm. (*D*) Probability histogram of the maximum side-lengths in 2D projections shows three clear peaks at positions that agree very well with the expectations of the model. (*E*) Distance errors as calculated by taking all the localization precision of each localization spot (*SI Appendix*, *SI Materials and Methods*). (*F*) Particles were classified into the 4 inherent classes (panel *C*, *SI Appendix*, Figs. S18 and S20) in order to determine their 3D reconstructions separately before merging them to form the full structure. The green, red, orange, and blue spheroids are the outcome of the 3D reconstruction algorithm for each class respectively, which were fitted into the theoretical volume (white clouds) that is accessible to the fluorophores after taking the dye linker into account (71). (*G*) Final reconstruction of the heptamer model (Movie S8). Purple spheroids are the final 3D reconstructions from all classes. (*H*) FSC analysis of the 4 reconstituted classes. Green, red, yellow, and blue curves represent class 1, 2, 3, and 4, respectively. The black curve is the half-bit criteria used to assess the resolution of the 3D reconstruction.

Fig. 5*A* shows a selection of experimentally measured particles with three fluorophores (see *SI Appendix*, Fig. S18 for additional projections). The histogram in Fig. 5*B* displays the spread of all measured distances from 179 such particles. Assuming a symmetric heptamer geometry of side length ~3.5 nm labeled with only three fluorophores, one expects four different classes with three distinct side-lengths of 3.5, 6, 7.5 nm (Fig. 5*C*). However, a robust assignment of distances is not possible because each side-length component can also appear shorter in a 2D projection, thus broadening its histogram component to lower values and causing overlaps between multiple side-lengths (*SI Appendix*, Fig. S19).

Interestingly, a clever solution can be implemented for identifying multiple side-lengths of a triangle that is situated in a randomly oriented plane. In the simplified case of an equilateral triangle of side length *d* regardless of orientation, the 2D projection of one of the side lengths will always fall in the range between d and $\frac{\sqrt{3}}{2}d$, thus, remaining close to *d* (*SI Appendix*, Fig. S19). This feature makes the distance *d* become pronounced in the 2D image plane. The situation is more complex for an arbitrary triangle, but the larger side length will always continue to be dominant, accompanied by a smaller tail. Fig. 5*D* presents the outcome of such an analysis when applied to the data included in Fig. 5*B*, revealing three clear peaks at 3.3, 5.5, and 7 nm, which match the geometry of αHL very well. The deviations in the measured distances are within our median localization precision of 0.7 nm (red line, Fig. 4*K*), which translates to a median distance uncertainty of 1.8 nm (Fig. 5*E*). We validated this result also by fitting the data from simulations (*SI Appendix*, Fig. S20).

To generate a 3D structural model of αHL from our experimentally resolved 2D projections of partially labeled complexes (Fig. 5*A*), a separation of multiple labeling configurations is required to avoid ensemble-averaging. To do so, we subjected the projections of particles with three fluorophores per PSF (*SI Appendix*, Fig. S18) to a supervised classification based on the resolved protein model (Fig. 4*G* and *SI Appendix*, Figs. S18 and S20 and *Supporting Text* 3). Here, the experimental projections were assigned to each class based on the matching score, ranging between 0 and 1. A particle was assigned to one of the classes if its score was the highest among the others. Then we selected the particles that scored above 0.8 in each class and reconstructed the 3D localization probability distribution of the fluorophore positions (Fig. 5*F*). We note that we arrived at this result using a pure 3D Gaussian model as an initial guess with no symmetry imposed (*SI Appendix*, Fig. S16). We fitted all the 3D reconstruction models into the theoretically accessible volume of the fluorophores attached to the C-terminal part of the protein and merged the 3D reconstructions together to build the complete heptamer structure of the protein complex (Fig. 5*G* and Movie S8). By computing the Fourier shell correlation (FSC) of the 3D reconstructions, we obtained a resolution of 6 Å for the reconstituted model (Fig. 5*H*). Again, fitting the distance distribution of the classified particles separately, indicates a good match with the heptamer model (*SI Appendix*, Fig. S20). These results highlight that the high precision of the method enables particle classification even when particles possess subtle differences.

### Resolving Membrane Proteins in Their Native Cellular Environment.

**Fig. 6.**
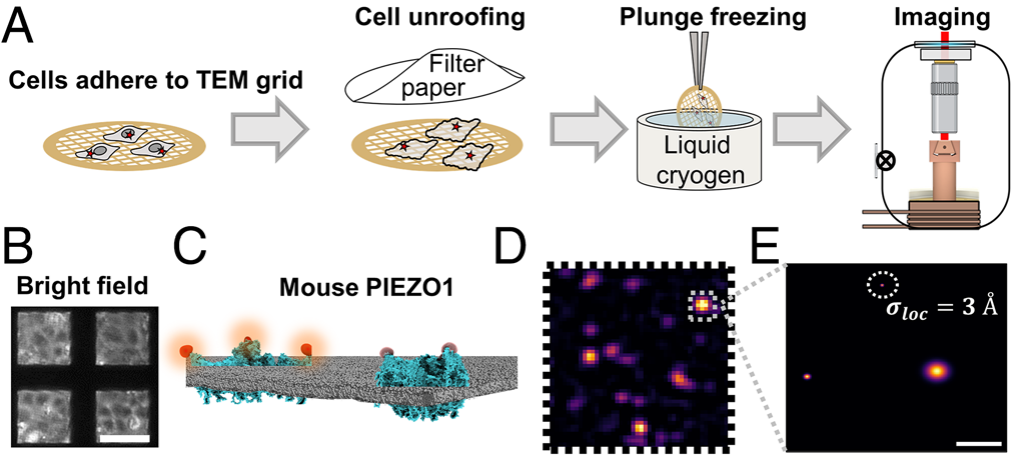
Imaging membrane proteins in their native cellular membrane. (*A*) Pipeline of the sample preparation: cells are first unroofing on a TEM grid, plunge frozen, and transferred to the microscope cryostat. (*B*) Bright-field image of mPIEZO1 transfected COS7 cells on a UF TEM grid before unroofing. (Scale bar is 90 µm.) (*C*) Schematics of fluorescence-labeled mPIEZO1 proteins in a cell membrane. (*D*) A fluorescence image (10 × 10 μm^2^) recorded by our cryogenic optical microscope from one of the mesh squares depicted in (*B*). (*E*) 2D image generated from a PSF with three polarization states reveals an mPIEZO1 conformation in which the extremities are separated by ~30 nm from each other (73). (Scale bar is 10 nm.)

The procedures described above can also be applied to intact cells and cell membranes. Here too, one can directly benefit from the protocols established in the Cryo-EM field for sample preparation. Given the thin slices that result in a typical realization, background autofluorescence remains manageable, as we reported very recently in two studies on membrane proteins (72, 73). In both experiments, we imaged transmembrane proteins in their native plasma membrane following an unroofing procedure (72, 74). As depicted in Fig. 6 *A*–*C*, this process isolates an intact cell membrane on a TEM grid by removing the upper portion of the cells. The sample is then plunge-frozen, transferred, and imaged as described in previous sections. This unroofing procedure was shown to maintain Ca^+2^-dependent exocytosis activity (74) and was more recently implemented in a Cryo-ET imaging pipeline, where it demonstrated retention of high-resolution structural information from proteins close to the cell membrane (75).

Fig. 6*D* presents an example of the fluorescence image obtained from the heterologous expressed mouse PIEZO1 protein (mPIEZO1), a mechanosensitive ion channel in COS7 cells. Fig. 6*E* shows an example, where one conformational snapshot of the protein was resolved within one diffraction-limited PSF, in native cellular membrane environment (73). In a second study, application of spCryo-LM as discussed in this work allowed us to image the clustering of endogenously expressed muscarinic acetylcholine receptor (M2R), a subfamily of the G-protein-coupled receptors (GPCRs), in native cardiac derived HL1 cells (72).

## Discussion and Outlook

In this work, we have demonstrated the promise of superresolution microscopy applied to a cryogenically fixed biological specimen, thus establishing spCryo-LM as a promising approach for structural biology at angstrom resolution. Indeed, two recent studies report on the applicability of our method to new discoveries in the conformational changes and clustering of membrane proteins (72, 73).

An interesting aspect of our methodology is its high throughput and robust statistical analysis which result from the high photon counts obtained from each fluorescent label under wide-field illumination. For example, in a single field of view (70 × 70 μm^2^), one can image about 5,000 particles within ~15 min if the sample can be labeled such that particles appear at densities ~0.5 to 1 per µm^2^, dictated by the optical diffraction limit. Several thousands of localization events per fluorophore in this period will give access to angstrom precision in the localization of individual fluorophores at a density of about one fluorophore per nm^2^ on each particle. This performance can be further enhanced. The angular space for polarization analysis can be doubled from [0 to 90°] to [0 to 180°] by adding a second polarization basis at a tilt of 45° (64). In addition, full 3D characterization of the dipole moments (76–79) would increase the capacity for identifying different polarization states. Furthermore, multiwavelength imaging would correspondingly increase the information density. Another exciting avenue for enhancing the number of localized molecules would be to exploit the narrow spectra at 8 K, enabling selective excitation and emission using narrowband lasers or emission filters.

We emphasize that our approach is based on cryogenics at liquid helium temperature, a platform that has not been commercially available. The two key advantages of using liquid helium for Cryo-LM are as follows: 1) Stronger laser illumination up to ~1 kW/cm^2^ can be applied without leading to devitrification. 2) The high laser intensity, together with enhanced blinking at liquid helium temperature, improves the photophysics in terms of collection efficiency for better localization and resolution below the diffraction limit. In fact, the strategy of shock-freezing and then transferring biological samples to a cryogenic microscope can also be extended to other forms of optical microscopy. In particular, suppression of dephasing in electronic transitions at LHe temperature will offer both larger cross sections and narrower spectra in Raman spectroscopy, ushering in label-free microscopy with molecular specificity (45, 80).

Being developed for shock-frozen samples on TEM grids, our work provides an efficient pipeline for correlative imaging, where angstrom resolution in both light and electron microscopy techniques can be achieved at the single particle level. Indeed, spCryo-LM offers some benefits that complement high-resolution implementations of electron microscopy. While the resolution of EM is intrinsically superior to light microscopy, the low electron contrast of biological matter often makes it challenging to resolve different domains of proteins, especially when considering limitations imposed by the acceptable level of electron dose (11, 16). The low contrast also brings about the need for averaging over many thousands of single particles and the use of template matching algorithms, e.g., as used in Cryo-ET (11). This feature poses a severe limitation on high-resolution investigations of proteins in their complex native environment due to a large background. Combination of electron microscopy with fluorescent labeling is, thus, highly desirable and, indeed, many efforts have already initiated correlative studies (37, 81). In this scheme, labeled particles would be prepared at relatively low densities to avoid having more than one particle per optical diffraction limit although advanced registration algorithms will likely provide solutions for higher densities. This line of correlative microscopy is currently in its early stages, leaving room for many future developments.

## Materials and Methods

### Cryogenic Sample Transfer.

We describe the whole sample transfer process in *SI Appendix*, including a step-by-step protocol. All information can be found in Figs. 1 and 2, *SI Appendix*, *Supporting Texts* 1 and 2 and Figs. S1–S4, and Movie S1.

### Preparation of Vitrified Fluorescent Molecules.

For single-molecule fluorescence measurements, 3.5 μL of 50 to 150 pM ATTO647N-maleimide fluorophores in a 25 mM HEPES buffer solution were loaded onto the TEM grid. The sample was then blotted after a 30-s incubation time using a Vitrobot (IV, Thermo Fisher) with standard Vitrobot blotting paper and parameters. See *SI Appendix*, *SI Materials and Methods*.

### Alpha-Hemolysin Labeling and Lipid Reconstitution.

C-terminal His-tagged αHL protein from *S. aureus* was purchased from Hölzel Diagnostika Handels GmbH (catalogue number CSB- YP639324FLFc7- 200). The protein was dissolved in 25 mM HEPES buffer, 25 mM KCl at pH 8 to a concentration of 10 µM. The protein was specifically labeled via the histidine linker on the C-terminal side of the protein with the dye HIS Lite™ iFluor™ 647 Tris NTA-Ni Complex which was purchased from AAT Bioquest (catalogue number 12618). To generate the protein lipid construct, we followed previously published protocols (82). See *SI Appendix*, *SI Materials and Methods*.

### Optical Setup.

Our experiments were conducted in a custom-built cryogenic microscope, which utilizes a Janis ST-500 flow cryostat to maintain liquid helium temperatures. The optical setup is similar to that used in our previous work, as described in refs. 46 and 58. See *SI Appendix*, *SI Materials and Methods*.

### Image Analysis.

We employed custom-written MATLAB software, as described in our previous works (46, 58), to analyze the raw image stacks from two polarization channels. See *SI Appendix*, *SI Materials and Methods*.

## Supplementary Material

Appendix 01 (PDF)

Movie S1. **Step-by-step transfer of vitrified samples at high vacuum and cryogenic temperature**. The video shows the entire sample transfer process. The video has been sped up 2 times to reduce its size.

Movie S2. **Breaking of the Carbon Mesh on the TEM Grid**. The video shows the carbon film tearing apart after laser illumination at an intensity of ~ 1 kW/cm^2^.

Movie S3. **Fluorescence imaging of ATTO647N in vitrified aqueous solution on carbon mesh TEM Grid**. The video was acquired at 70 Hz using one camera after polarization splitting.

Movie S4. **Fluorescence imaging of ATTO647N in vitrified aqueous solution on UltrAuFoil TEM grid**. The video was recorded on one of the cameras after polarization splitting at 70 Hz.

Movie S5. **Fluorescence imaging of smURFP in vitrified aqueous solution on UltrAuFoil TEM grid**. The data was recorded on one of the cameras after polarization splitting at 70 Hz.

Movie S6. **Fluorescence imaging of DNA nanoruler (30 nm, tilibit nanosystems GmbH) in vitrified aqueous solution on UltrAuFoil TEM grid**. The data was recorded on one of the cameras after polarization splitting at 70 Hz.

Movie S7. **Fluorescence imaging of αHL sample reconstituted in SUV on UltrAuFoil TEM grid**. Fluorescence images of vitrified αHL reconstituted in SUVs after incubation on UltrAuFoil TEM grid with 2 nm carbon on top. The video shows sparse point spread functions with good on-off ratio. Data were recorded at 70 Hz using one camera after polarization splitting.

Movie S8. **3D reconstruction of αHL sample**. The video shows the final 3D reconstruction of each αHL class labelled with three fluorescent molecules. The 3D reconstructions were then merged to generate the full heptameric assembly. Scale bar is 10 nm.

## Data Availability

All study data are included in the article and/or supporting information. The data presented in Figs. 2-5 are provided as Source Data (83).
