# Supplementary material for: Cryogenic light microscopy of vitrified samples with angstrom precision
Source: Proc Natl Acad Sci U S A. 2025 Dec 3;122(49):e2513583122. doi: 10.1073/pnas.2513583122 (PMC12704754; doi:10.1073/pnas.2513583122)
Supplement: Supplementary file 1 — Appendix 01 (PDF) [file pnas.2513583122.sapp.pdf]

Supporting Information for

Cryogenic light microscopy of vitrified samples with angstrom precision

Hisham Mazal[1,2], Franz-Ferdinand Wieser[1,2,3], Daniel Bollschweiler[4], and Vahid Sandoghdar[1,2,3]

1. Max Planck Institute for the Science of Light, 91058 Erlangen, Germany
2. Max-Planck-Zentrum für Physik und Medizin, 91058 Erlangen, Germany
3. Department of Physics, Friedrich-Alexander University of Erlangen-Nürnberg, 91058 Erlangen, Germany
4. Max Planck Institute of Biochemistry, Planegg, Germany

Correspondence to: Vahid Sandoghdar

Email: vahid.sandoghdar@mpl.mpg.de

This PDF file includes:

Supporting text 1 to 3

Figures S1 to S20

SI Materials and Methods

Supporting protocol

Legends for Movies S1 to S8

SI References

Other supporting materials for this manuscript include the following:

Movies S1 to S8

Supporting text

1. **Cryogenic high-vacuum transfer**

The design features of the transfer system are primarily dictated by temperature and contamination requirements. Specifically, the plunge-frozen grid must be maintained below 130 K and protected from direct exposure to air. In traditional cryo-electron microcopy (Cryo-EM) holders, the grid is loaded under liquid nitrogen and enclosed by a cold shield as it briefly traverses through air before insertion into the microscope. Residual contamination in the form of small ice crystals is sometimes observed in this process. A direct transition from liquid nitrogen to vacuum has proven to significantly reduce contamination, a critical consideration in correlative studies involving multiple transfers of a grid between different instruments (1). We adapted this strategy in our cryogenic optical microscope (2, 3) with a series of modifications as depicted in Figs. S1-S4.

First, plunge-frozen grids are mounted on a purpose-built cartridge (Fig. S2) inside a dedicated preparation chamber as depicted in Fig. S1A. The preparation chamber includes a working platform that holds a cold stage (cartridge holder) and a TEM grid box groove. A glass vessel (SCH 9, KGW Isotherm, ~ 120 ml) serves as a liquid nitrogen (LN) container, and a vertical vacuum manipulator is used to lower or raise the glass vessel. The chamber is initially purged with dry nitrogen to reduce water vapor. The glass vessel is then filled with LN and is moved upward to cover the working platform completely. In this case the TEM grid can be picked up and mounted safely onto the sample cartridge in LN. The grid is securely held in place by a clamp with a thin profile to allow positioning it under a long working distance microscope objective (Mitutoyo Plan Apo HR, 100x, 0.9 NA) in the cryostat. To ensure proper surface contact between the sample cartridge and the cold stage, we used two repelling magnets press-fitted onto the both components (see Fig. S2 A-B)

The high-vacuum transfer shuttle (Pfeiffer, 420MDM040-0500) consisted of a linear manipulator and a cooling stage as core components (Fig. S1E). The linear manipulator with 500 mm range and maximum torque of 2.3 Nm is connected to a conical shaped copper head that allows sample cartridge loading via a small screw. The high-vacuum shuttle is evacuated to ~10^{-6} mbar and cooled to LN temperature. The cold stage is actively cooled during the whole transfer process, maintaining a stable temperature below the devitrification point (Fig. S1G). High-vacuum is maintained within the shuttle by the cold stage after closing the gate valve to the pump. The shuttle interfaces seamlessly with the microscope through a KF50 flange. The external interfaces are flexible such that the design can easily be adapted to suit other microscopes.

Upon loading the TEM-grid we lower the LN vessel to provide side access to the cartridge and begin evacuating the chamber. As some LN remains in the glass vessel, the vacuum level reaches 10^{1} -10^{-1} mbar, depending on the LN level. However, we minimize condensation by extensively purging the preparation chamber with dry nitrogen throughout the entire process until evacuation begins. This reduces the water vapor level in the chamber. However, based on Cryo-EM inspections we found that such water vapor still exists and condensates onto our TEM grid. In order to avoid these condensations, one can install a mechanical shutter on the sample cartridge to protect the TEM grid while transferring in and out of the preparation chamber (see Fig. 2 in the main manuscript, and Fig. S6-S7). This is indeed commonly practiced in EM transfer systems. In fact, one can exploit the rotation of the manipulator around its axis to release a weakly bound cover. This allows an unbound cover to be placed on top of the cartridge. Once the cartridge is picked up by the transfer shuttle and a high vacuum level is reached, the cover can be released by rotating the cartridge upside down before insertion into the optical microscope. Next, we open the high-vacuum shuttle's gate valve and use the manipulator to load the sample cartridge. Once loaded, the manipulator is retracted and parked on the cold stage to maintain the sample cartridge at LN temperature. Temperature readings of the cartridge before and after the loading step remain stable close to LN temperature (Fig. S1D,G).

Then, we close the preparation chamber gate valve and allow the transfer shuttle to establish high vacuum level again (10^{-6} mbar). After reestablishing high vacuum in the transfer shuttle, we close its gate valve and vent the connection interface to disconnect it from the preparation chamber. The transfer shuttle is then connected to the cryogenic optical microscope. After establishing high vacuum level (10^{-6} mbar) at the connection interface, we insert the sample cartridge into the microscope's cold finger. The optical microscope is pre-evacuated to 10^{-6} mbar and precooled to liquid helium (LHe) temperature prior to sample insertion. After inserting the sample into the microscope, thermal contact with the cold

stage adapter is sustained through the side faces of the dovetailed piece, facilitated by two pairs of repelling magnets embedded in the cartridge and the cold stage adapter. Pogo pins connect the temperature sensor of the sample cartridge to the controller (Fig. S3). Temperature readings from silicon diodes on the cartridge and the cryostat show 8.3 K and 4.3 K, respectively (Fig. S3).

The optical microscope (Fig. S3) is built around a Janis-500 cryostat, which is connected to an extended cold finger with a complementary shape to the sample cartridge. The objective is mounted from the top and has a working distance of 2 mm and numerical aperture (NA) of 0.9. The objective can be positioned against the sample with an x, y, z piezoelectric scan stage. The laser beam is guided through a window to reach the objective and is aligned at the center of the TEM grid (Fig. S3). We have tested the stability of the optical microscope and found a mechanical drift in the order of ~ 5 nm/min (Fig. S3F). See Fig. S3E for the complete optical scheme of the microscope, and Fig. S4 for a real picture of the system

See supporting protocol for a step-by-step procedure, and Movie S1.

2. Validation of vitreous ice stability upon laser illumination.

To obtain a sufficiently large number of photons during the on-time blinking of a single fluorophore, it is desirable to excite the molecule at a high rate, leading to laser intensities in the range of 0.3 - 1 kW/cm^2. However, such high light intensities are known to cause severe sample devitrification at 77 K (4, 5), and film deformation especially in the case of holey support film (carbon) TEM grids (see Fig. S5). Gold TEM grids were found to handle high optical intensities (~ 0.6 kW/cm^2), to possess superior mechanical stability and to maintain stable vitreous ice condition (4, 6, 7). To verify this experimentally, we vitrified aqueous samples on several UltrAufoil R2/2 200 mesh (S373-7-UAUF) TEM grids and exposed each one of them to varying laser intensities in the cryostat for few hours. The samples were subsequently retracted from the cryostat and investigated by Cryo-EM imaging (Fig. S6-S7) to examine the presence of devitrified ice.

For this, we used a vitrified fluorescent aqueous solution because in the case of pure water, the holes would appear dark, making it impossible to distinguish the presence of water using an optical microscope. Thus, it would not be easy to asses if the grids were over-blotted. In the case of a fluorescent solution, the signal should be visible across the grid (see Fig. 2B in main text).

We used TEM grids made of gold (UltrAufoil R2/2 200 mesh, S373-7-UAUF), which were plasma-cleaned at 24% plasma power for 60 seconds in a Faraday cage (Diener, Pico 500 W). 3.5 µL of 10-20 nM iFlour647 Tris NTA chelator solution in a buffer composed of 50 mM HEPES, 1% glycerol, and 0.01% TWEEN 20 was applied to the grid and allowed to spread for 30 seconds. The sample was then blotted using the following parameters: 2 seconds blotting time, -2 blotting force, and 0 waiting time. Next, the sample was transferred into our optical microscope following the procedure outlined in the previous section and was allowed to equilibrate for 1–2 hours.

The samples were illuminated with a 645 nm laser source in wide-field mode at different laser intensities (0, 0.16, 0.34, 0.65, 1 kW/cm^2). First, we quickly screened the grid area (each field was exposed for ~5 seconds) until we reached the center of the TEM grid. We then illuminated each area around the center intensively for 2–10 minutes, corresponding to a typical single-molecule acquisition time discussed in the main manuscript. We used the internal marker on the TEM grid to navigate across the grid. Our field of view was 512 x 512 pixels, with a pixel size of 227 nm, which corresponds to a complete grid mesh size of ~100 µm x 100 µm (see Fig. 2B). In this case, we illuminated a 6 x 6 region around the center of the grid, while the rest was illuminated for a short exposure time of a few seconds. The fluorescence signal shown in Fig. 2B was used to optically confirm the presence of vitreous ice on the sample. The bright spots/regions indicate the presence of the fluorescent solution on the grid, serving as a validation before the subsequent Cryo-EM measurement. Each laser intensity was measured in duplicates/triplicates (2–3 grids each).

At the end of the measurements, the samples were retracted and immediately stored in a LN storage dewar. They were then transferred to the Cryo-EM facility in Munich for vitreous ice assessment and analyzed as explained in Methods.

All grids survived laser illumination and showed vitreous ice conditions, except for one sample (sample number 5), which exhibited some crystalline ice patterns (see Fig. S7). We attribute this to bad handling of this particular sample during a transfer process.

3. Particle classification

We used a supervised classification approach for particle classification. To do so, we generated a library of images based on the structural model of αHL (PDB: 7hal). For each of the four classes we rotated the particles in 3D and then projected the image onto the 2D plane. We used a rotation increment of 5° for x, y and z axes. Next, we cross-correlated the experimental image to all the library generated from all the classes, and determined the maximum cross-correlation value (score), ranging from 0 to 1. The class that yielded the highest score is assigned to the experimental image.

To test the accuracy of this approach, we generated random images from all four classes. Here, we used the center position of the fluorophore exactly as in the structural model but with a different localization precision. Then we subjected these images to 2D cross-correlation as explained above. As depicted in Fig. S19A, if we assume high localization precision 7 Å (similar to our experimental data), the classification reaches 100% accuracy. This also holds for 1 nm localization precision (Fig. S19B). However, if we increase the localization precision to ~ 2 nm, ~ 20% of class 1 and class 2 are misclassified (Fig. S19C). Next, we simulated data with random centre position displacement of ± 1 nm, assuming 7 Å localization precision. Analysis of particle classification under these conditions revealed substantially increased misclassification rates, particularly for classes with shorter distances such as class 1 and class 2 (Fig. S19D). Thus, our conclusion is that the lower the localization precision, the larger the misclassification. As a result, if one seeks to obtain informative data for structural biology, one must reach localization precision better than 2 nm.

Supporting figures

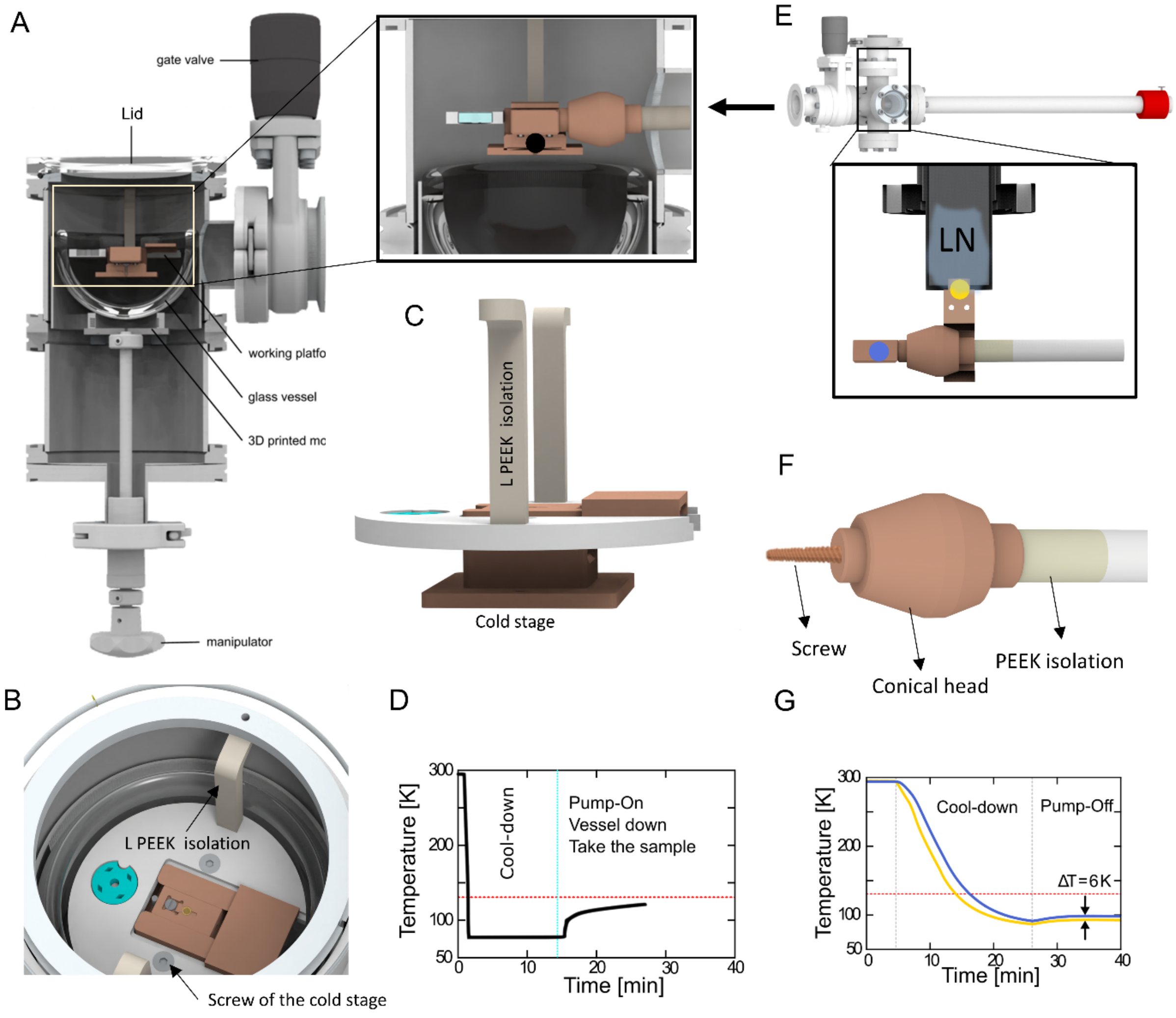

Fig. S1. Sample loading chamber. **(A)** Cut view of the sample loading chamber. An insulated glass vessel (KGW Isotherm) holding up to 120 ml of liquid nitrogen is attached to a vacuum manipulator for vertical movement. In the fully lifted position, the vessel allows complete immersion of the working platform in liquid nitrogen. The TEM grid can be mounted onto the cartridge with a pre-cooled tweezer without the risk of de-vitrification or contamination. The loading chamber is sealed on top with an acrylic plate, connected to a nitrogen gas supply line in the back, and to a pumping station (Pfeiffer) via a gate valve on the side. **Inset**, When the vessel is fully retracted, the transfer manipulator can access the cartridge from the side. **(B)** Shows the working platform hanging from two polyether ether ketone (PEEK) isolation L-pieces mounted on the top flange. **(C)** Shows a side view of the working platform, featuring a copper cold stage suspended from the platform by two mounting screws. **(D)** Temperature readout of the sensor placed on the cold stage (black circle, inset of (A)) during the transfer process as describe in Supporting text 1. The temperature remains below the devitrification point. **(E)** High-vacuum shuttle after loading and transfer of the sample cartridge, where the cartridge brought in contact with the cold stage to keep the sample below the devitrification point. **(F)** Linear manipulator with the large-mass conical head. The tip of the manipulator is extended with 20 mm poly ether ketone (PEEK) isolation. The conical head contain a screw for loading the sample cartridge via its thread (see Fig. S2B). **(G)** The temperature readouts of the sample cartridge (blue circle in C) and the cold stage (yellow circle in C). The temperature is stable and remains below the devitrification point. Color code is similar to the inset of E.

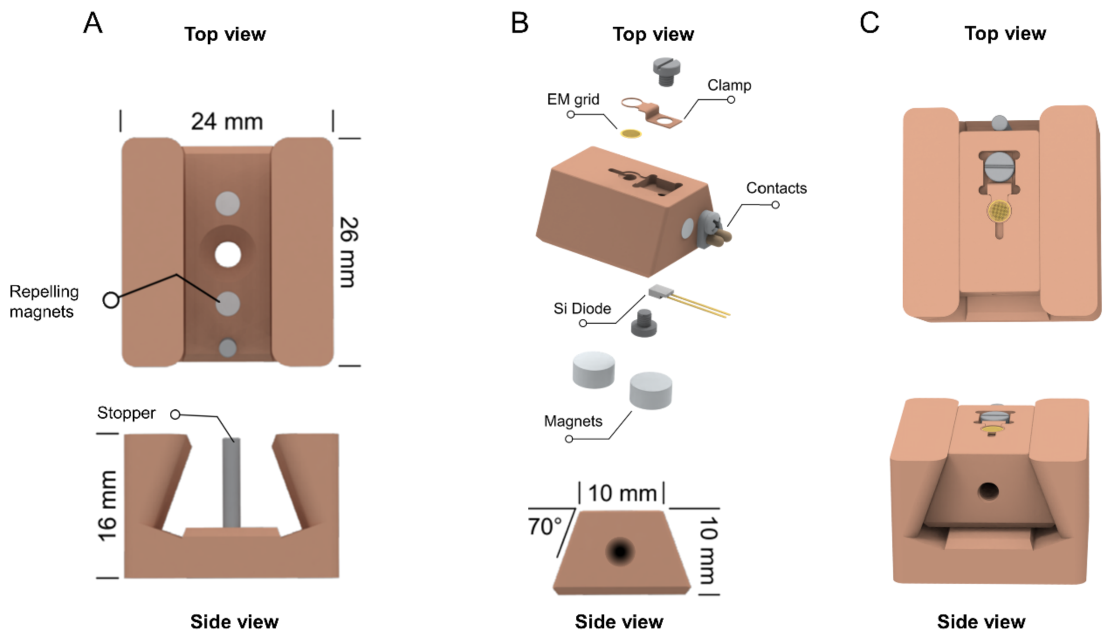

Fig. S2. Sample cartridge and cold stage adapter. (A) Top and side views of the cold stage, designed with a complementary dovetail-shaped structure. It contains two repelling magnets and a stopper to properly position the sample cartridge. **(B)** View of the sample cartridge. A single TEM grid is mounted with a small clamp and a screw. The dovetail-shaped cartridge inserts into the cold stage adapter and is held in place by two pairs of repelling magnets and an end-stop. A bare silicon diode temperature sensor (DT670) is integrated into the bottom of the cartridge. **(C)** Final assembly of the parts in (A) and (B). The cold stage in (B) is similar to that used in the optical microscope but includes two additional pogo-pins for temperature readout from the sensor integrated into the sample cartridge, see Fig. S3.

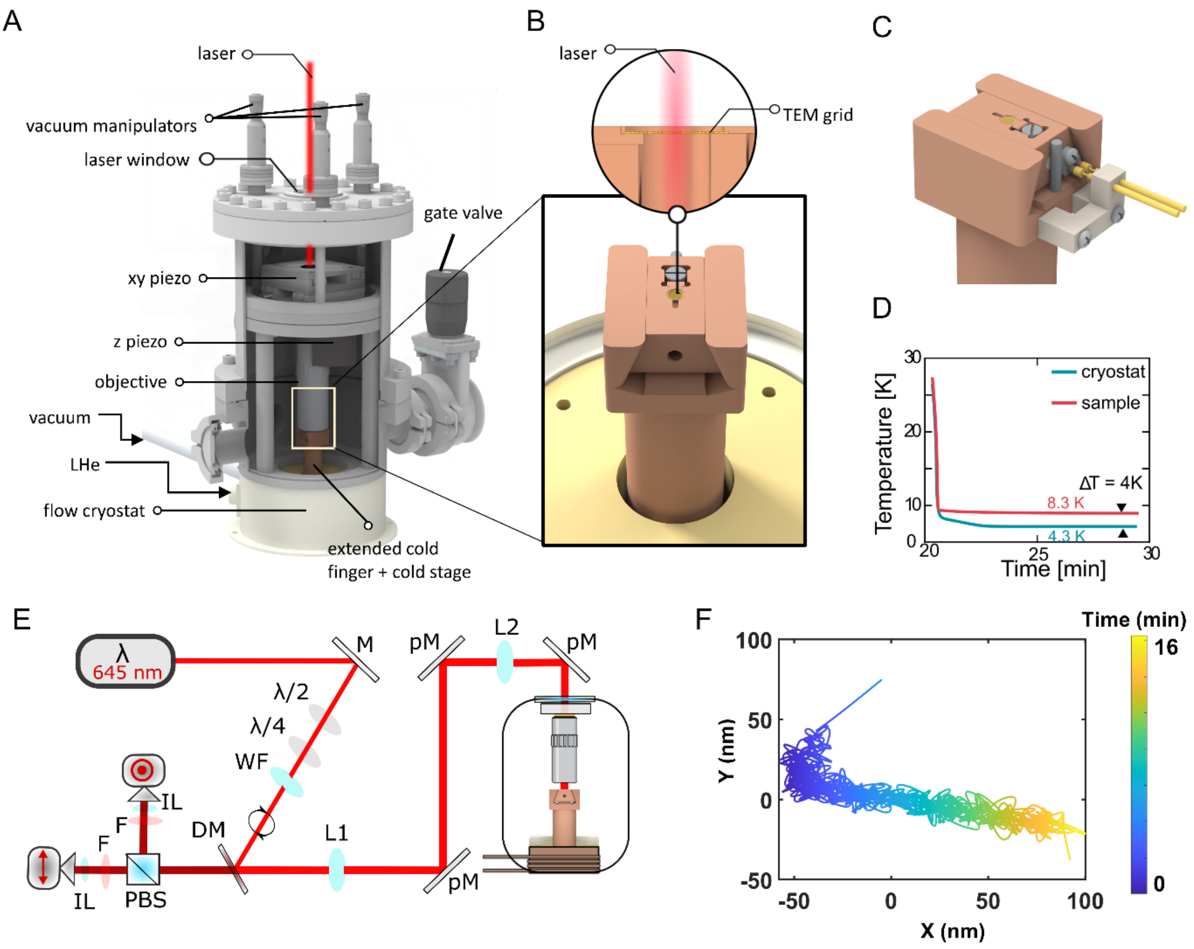

Fig. S3. Modified high-NA cryogenic microscope. (A) View of the cryostat and vacuum system of the optical microscope. The microscope is built around a liquid helium cryostat (Janis-500) shown in the lower part. An extension chamber with four KF40 ports allows access from the side via a gate valve. The cryostat cold finger is extended to place the cold stage adapter on the axis of the vacuum port for inserting the cartridge. An objective (Mitutoyo Plan Apo HR, 100x, 0.9 NA) connected to an x-y-z piezo stage allows sample scanning and focusing. **(B)** Close-up and side views of the sample cartridge loaded into the cold stage adapter inside the microscope. The view is from the side of the transfer shuttle access and the gate valve indicated in (A). Inset shows a side view of the optical axis. The laser beam traverses the sample through a hole in the cartridge. **(C)** Rear view of the cold stage of the image in (B), depicting the connection between the sample cartridge and the pogo pins assembled on the cold stage for temperature readout. **(D)** Temperature readout indicates the sample cartridge stabilized at ~ 8 K. (**E**) Schematics of the optical microscope pathway, which allows us to select the polarization of the fluorophores in our samples as explained in detail in Ref (2). A linearly polarized laser beam (645 nm) is transformed to a circularly polarized beam using λ/2 and λ/4 waveplates. A wide-field (WF) lens is inserted to focus the laser beam onto the back focal plane of the objective. L1 and L2 establish a telecentric lens system with 400 mm focal length. The polarization of the laser is maintained along the path using polarization-maintaining coated mirrors (pM). The fluorescence emission is split using a dichroic mirror (DM), and the polarization is split using a polarized beam splitter (PBS). The emission is then filtered using an emission filter (F) and focused onto an EMCCD camera using an imaging lens (IL). The microscope is evacuated up $1x10^{-6}$ mbar and the cold stage is cooled down to 4 K using liquid Helium. Once the sample inserted, we turn off the vacuum pump and allow the system to relax for 1-2 h before we start acquiring data. **(F)** The mechanical stability of our microscope is very high, as we achieve a drift of ~ 5 nm/min, close to the manufacturer value of ~ 2 nm/min.

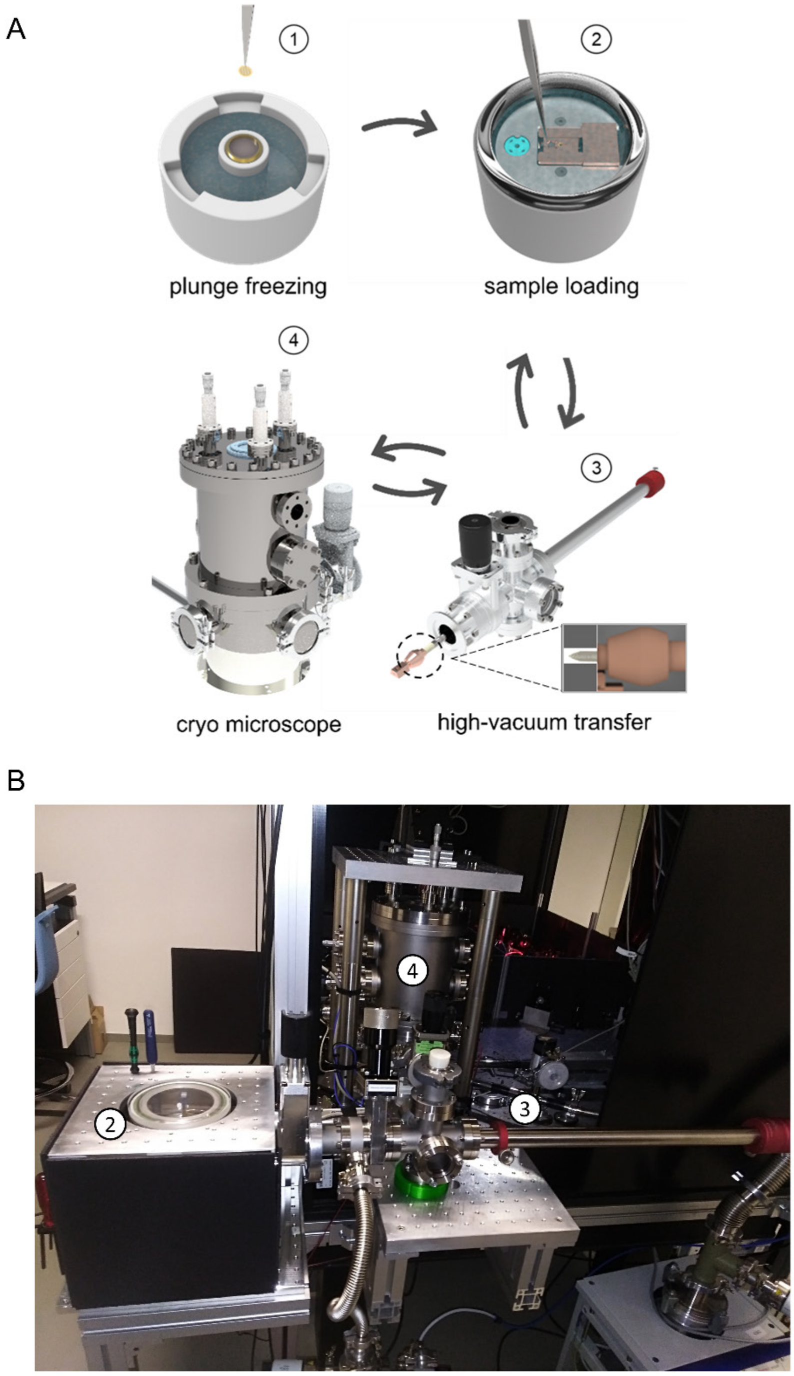

Fig. S4. Cryogenic high-vacuum transport shuttle. (A) Overview of the sample transfer scheme. The sample is plunged into liquid ethane (1) and then loaded onto the sample cartridge inside a liquid nitrogen LN bath in a special preparation chamber (2). A high-vacuum transfer shuttle (3) is used to move the sample from the preparation chamber to the optical microscope (4). This process can also be executed in a reverse order for correlative studies. **(B)** Real image of the setup showing the preparation chamber (2), high-vacuum transfer shuttle (3), and the optical microscope (4).

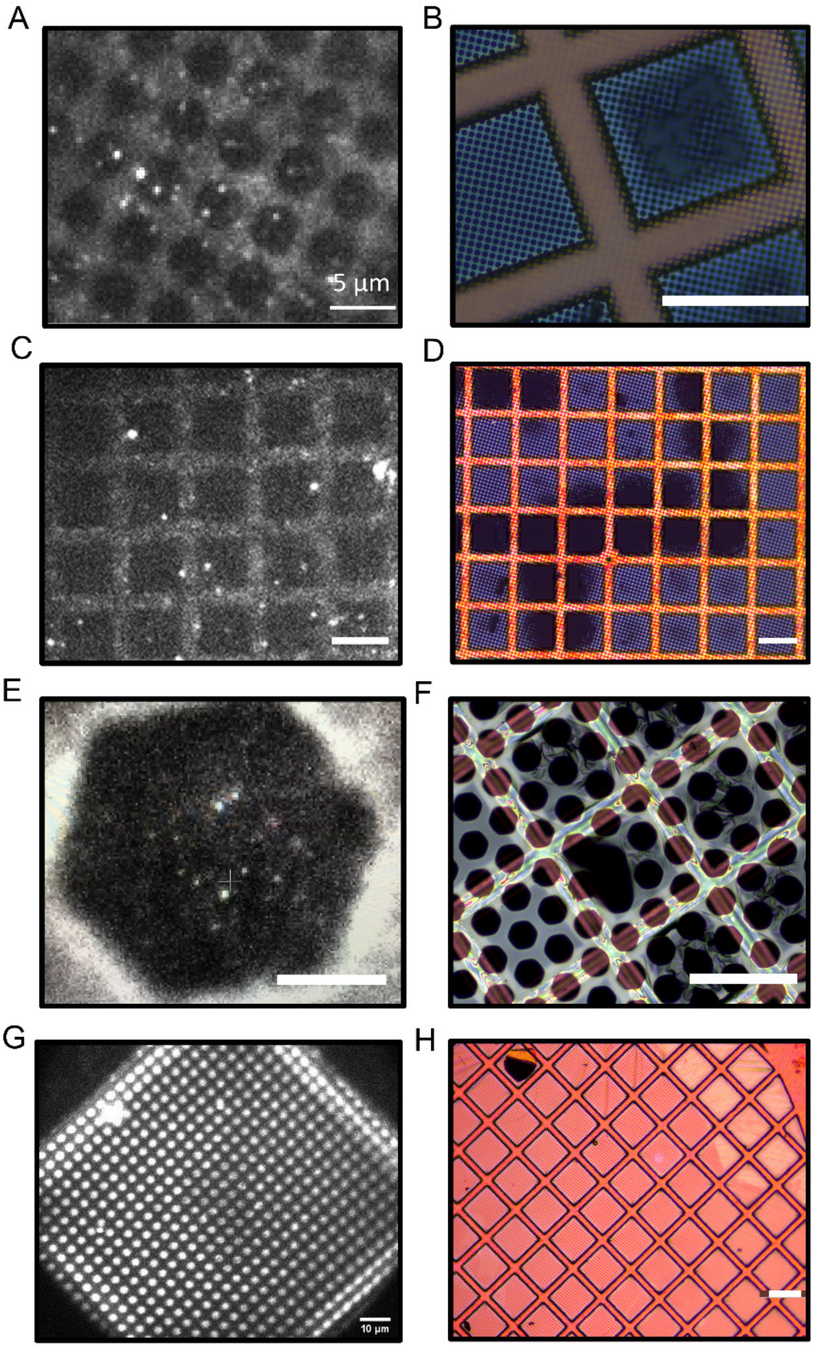

Fig. S5. TEM grid mesh stability. Aqueous solutions (25 mM HEPES and 150 pM ATTO647N) were vitrified on different carbon mesh grids TEM grids. The samples were loaded into the microscope and illuminated with a laser intensity of ~ 0.65 kW/cm^2 for fluorescence images recordings. Afterwards, the sample was retracted and inspected visually using a bright-field microscope. **(A)** Fluorescence image of a holey support film (R3.5, 200 mesh, S177-7 Plano-GmbH). Bright spots are single fluorescent molecules, and the low intensity grey background is the holey support film. **(B)** Bright-filed inspection after the recording. The data show that some carbon films got damaged. Brown bars are the gold framework, and the bluish mesh is the carbon film. Scale bar is 100 µm. **(C-D)** Similar to (A-B), but for square holey support film (R7/2 square 200 mesh, S117-7 Plano-GmbH). Scale bar is 100 µm. **(E-F)** Similar to (A-B) but for hexagonal holey support film (R25/15 hexagonal 200 mesh, Hex15-7 Plano-GmbH). The image in (E) shows one hexagonal hole (black region) filled with single fluorescent

molecules (bright spots). The carbon film is shown on the periphery with high intensity due to high reflection. Scale bar in E is 10 µm and in F is 100 µm. **(G-H)** Similar to (A-B) but for gold mesh TEM grid (UltrAuFoil 200 mesh, QF R2/2 + 2nm carbon film on top, S373-7-UAUF-2C, Plano GmbH), which is the only grid that remained intact and stable. The bright regions in (G) are holes, containing vitrified aqueous solution with 50 nM fluorescent molecules. The black/grey mesh is the gold mesh support. Scale bar in G is 10 µm and in H is 100 µm. (see associated Movies S1-3).

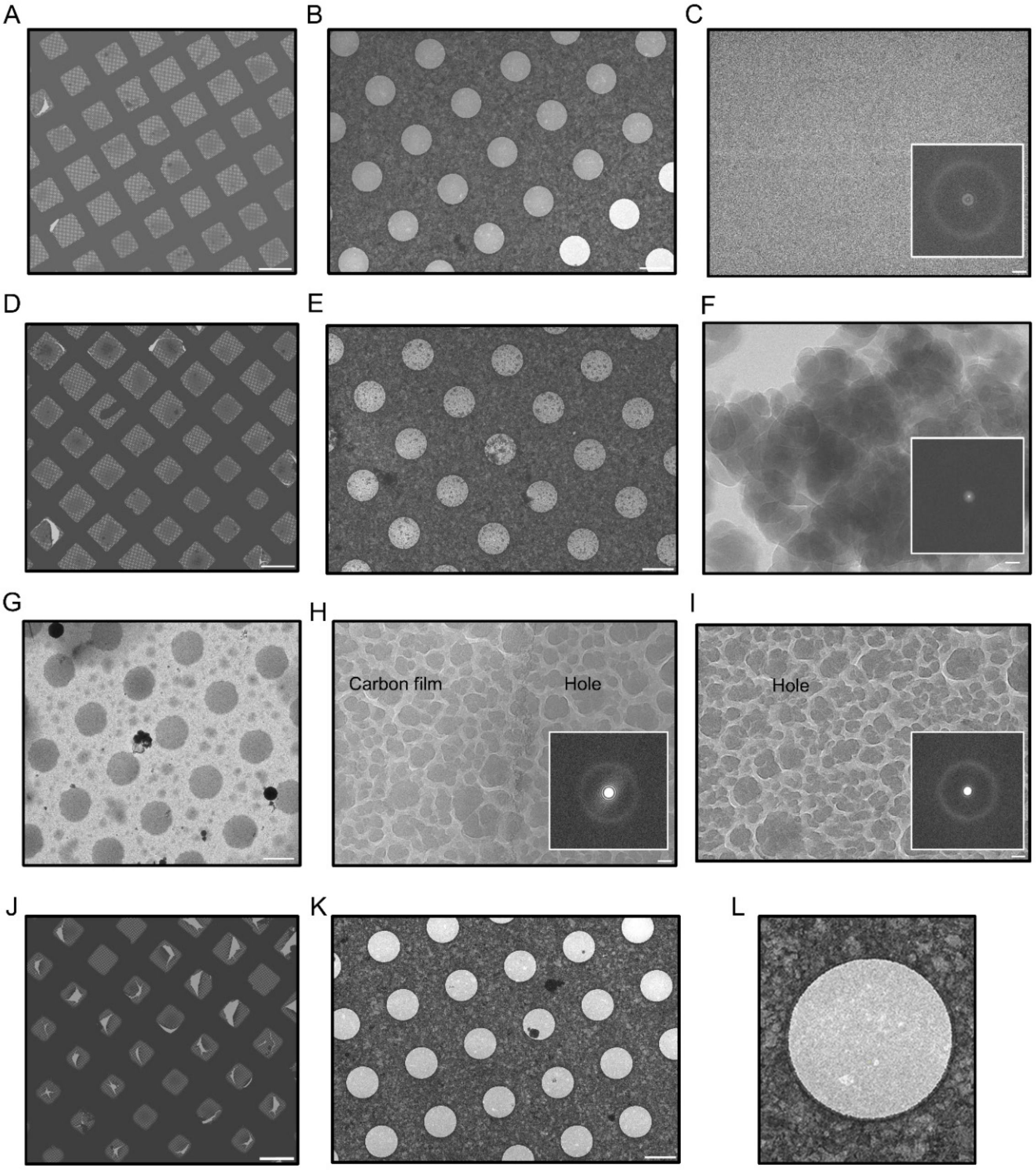

Fig. S6. Assessment of TEM grid cleanliness throughout the transfer procedure. Cryo-EM was used to evaluate contamination levels on TEM grids at key checkpoints. **(A-C)** Contamination assessment at the initial loading step, where the grid was clamped inside the preparation chamber. **(A)** UltrAuFoil TEM-grid atlas. Scale bar, 100 µm. **(B)** Zoomed-in view of a grid square. Scale bar, 2 µm. **(C)** High-magnification view inside a hole. The inset shows a power spectrum, indicating intact vitreous ice. Scale bar, 20 nm. **(D-F)** Grid status after transfer and imaging at 0.65 kW/cm². **(D)** UltrAuFoil TEM-grid atlas. Scale bar, 100 µm. **(E)** Zoomed-in view showing sparse, fine-grade contamination covering the holes. This contamination is likely due to water vapor freezing onto the grid. Scale bar, 2 µm. **(F)** High-magnification view inside a hole, focusing on the contamination. The power spectrum in the inset confirms the presence of intact vitreous ice. Scale bar, 20 nm. Similar patterns were also observed in different systems (7, 8). **(G-I)** Contamination source assessment using holy support film (CF-2/2-2AU-50). **(G)** Low-magnification image showing contamination covering the entire grid. Scale bar, 2 µm. **(H)** Carbon film-hole interface showing a continuous film of contamination. **(I)** High-magnification view inside a hole showing a high level of contamination, likely from fine water vapor in the preparation chamber condensing on the cold sample cartridge. Scale bars in (H) and (I), 20 nm. **(J-L)** Grid status after transfer and imaging at 0.65 kW/cm² using a 100 µm pinhole cover on top. **(J)** UltrAuFoil Grid

atlas. Scale bar, 100 µm. **(K)** Zoomed-in view shows a very clean TEM grid. Scale bar, 2 µm. **(L)** High-magnification view inside a hole, showing clean, intact vitreous ice suitable for correlative Cryo-EM measurements. This result also confirms that the contamination seen on the TEM grids is surface contamination from water vapor inside the preparation chamber. Hole diameter is 2 µm.

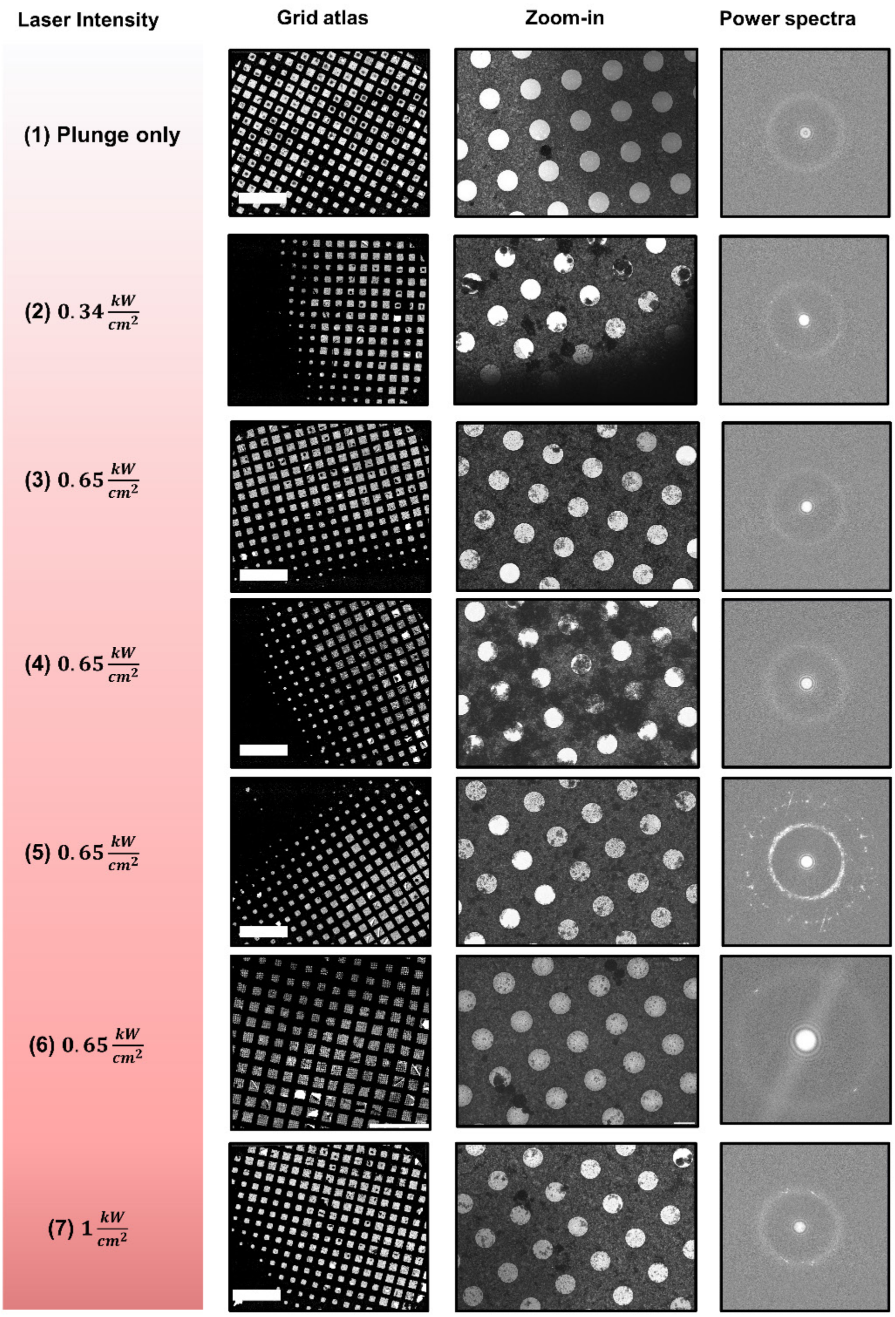

Fig. S7. Vitreous ice stability as a function of laser irradiation. Cryo-EM analysis of TEM grids exposed to various laser intensities at a wavelength of 645 nm, as indicated on the left-hand side of each row (1-7). Micrographs of each TEM grid pattern (left column), the holey gold foil at intermediate magnification (middle column), and the associated power spectra (right column) of vitreous ice exposures in holes at high magnification (105k×, equivalent to 0.85 Å/px). The scale bar of the left column is 500 µm, and the hole size in the middle column is 2 µm. No notable devitrification was observed at any of the laser intensities, except for sample (5) due to bad handling. These experimental results match the theoretical calculation of a laser-devitrification threshold of ~1 kW/cm² for a holey gold film at a 645 nm wavelength under vacuum (7). Some crystalline ice formation could be observed at various locations of the grids, independently of the laser exposure state. This is most likely due to the presence of transfer ice that accumulated during different steps (dewar transfer, grid clipping process, microscope transfer) of sample preparation.

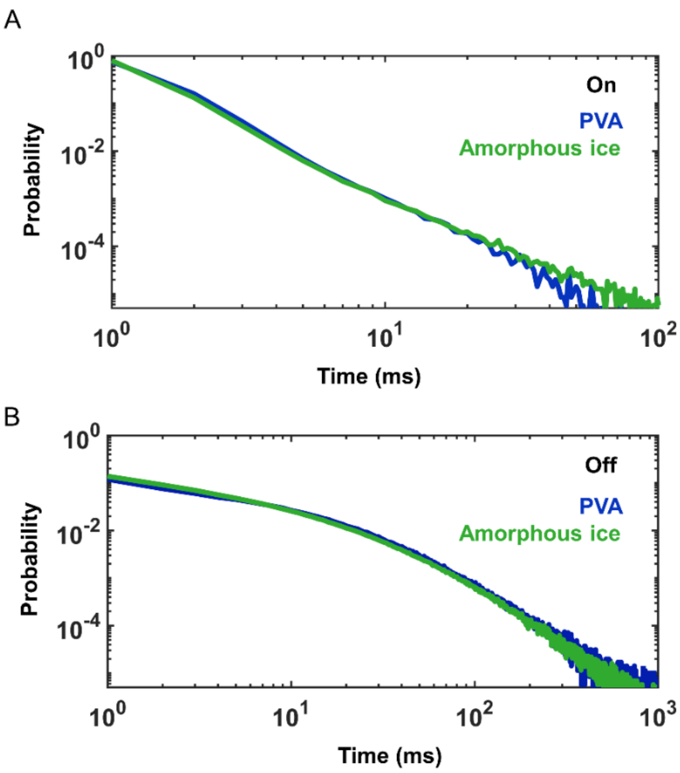

Fig. S8. Comparative photophysical analysis of ATTO647N in polyvinyl alcohol (PVA) versus vitreous ice matrices. (A) On-times distribution of ATTO647N in PVA (blue curve) compared to that in vitreous ice (green curve) at high vacuum and LHe temperature. **(B)** Off-times distribution. Color code and condition is the same as indicated in (A).

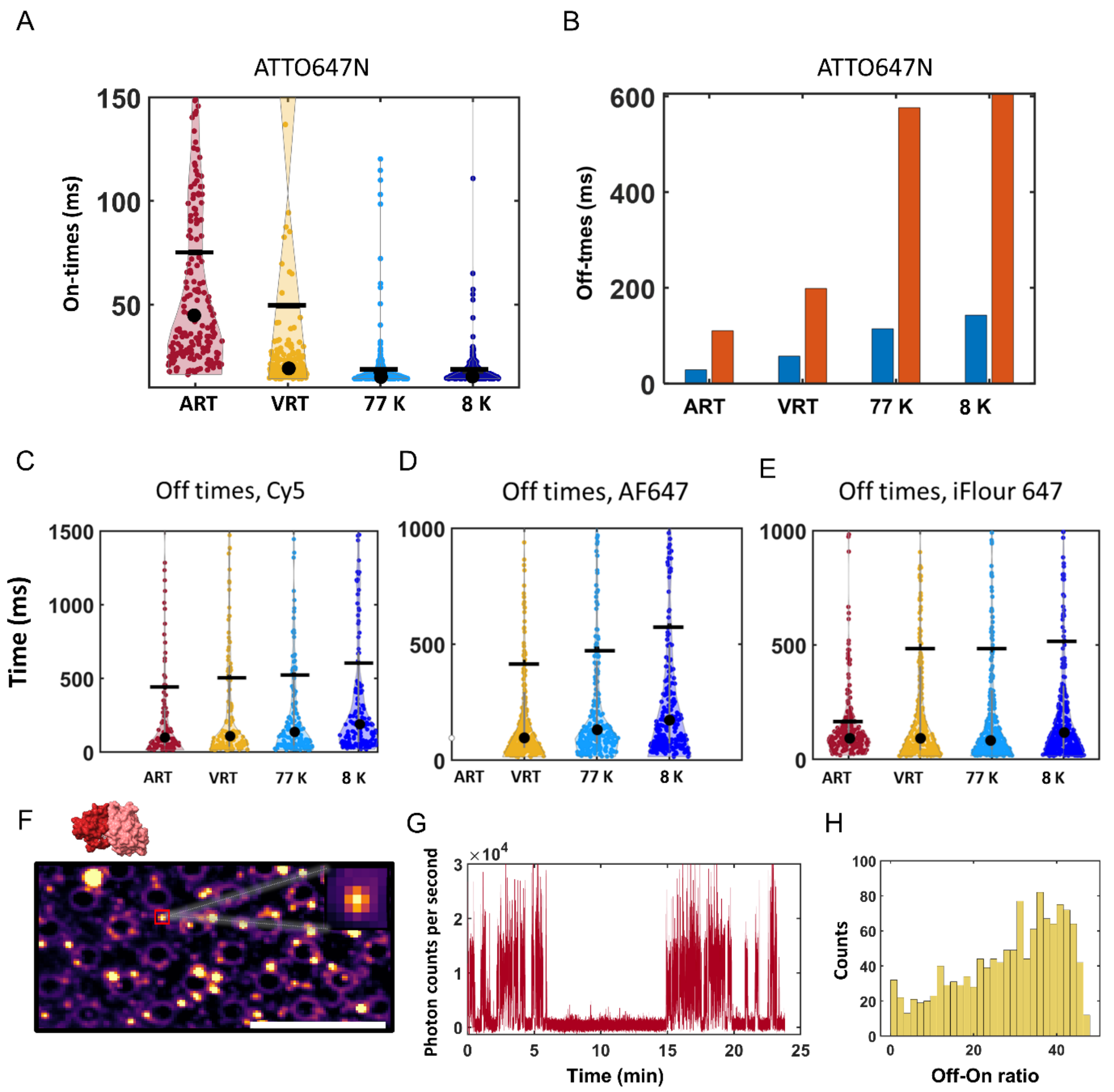

Fig. S9. Photophysics characterization. (A) Distribution of on dwell-times for ATTO647N recoded at different conditions (atmospheric room temperature (ART), vacuum RT (VRT), Vacuum 77 K, and Vacuum 4 K). **(B)** Comparing two different calculation approaches for the median off-time distribution. The orange bar represents the median of the mean off-times from each molecule, and the blue bar represents the median calculated directly from all off-time values. The results indicate a locally heterogeneous environment that affects the photoblinking behavior. (**C-E)** Distribution of off dwell-times for different organic fluorophores**:** Cy5 (C), AF647 (D), iFlour 647 (D). ART in (D) was not measured due to fast photobleaching of the sample. Black line indicates the mean values, and black circles indicate the medians. **(F-H)** smURFP protein imaged in vitreous ice. **(F)** Image of smURFP protein detected on UF TEM grid. Inset show the smURFP protein dimer (PDB: 6fzn) structure, and exemplary points spread function (PSF) (from a sum of 1000 frames). Scale bar is 10 µm. **(G)** Intensity time trace shows exceptionally long off-times. **(H)** Off-on ratio histogram as obtained from many smURFP molecules.

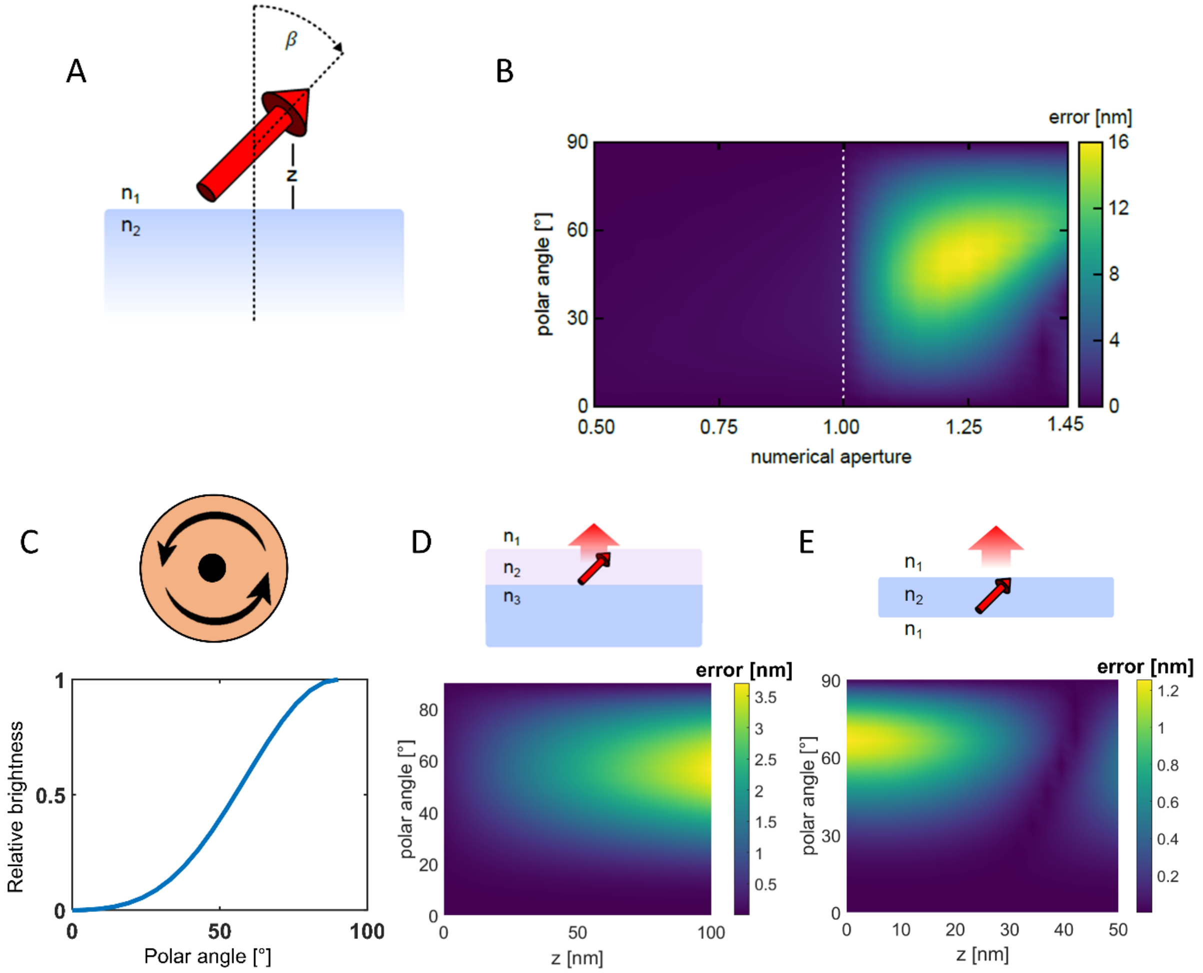

Fig. S10. Localization error due to dipole orientation. (A) Schematics of a dipole with orientation β ∈ [0, 90◦] and distance from the interface (z). The localization error is calculated from a simulated point-spread function, which is numerically computed for polar angles and an emission wavelength of 660 nm under various sample configurations. **(B)** Localization error for a dipole at a glass/vacuum interface as a function of the polar angle and numerical aperture, where the dipole is placed in vacuum. The refractive indices are n_1 = 1 and n_2 = 1.52, the pixel size is set at 100 nm, the thickness of each layer is set to infinity, and light is collected with an immersion objective through the glass substrate. The deviation from the true position is less than 0.5 nm for NA<1. **(C)** Excitation of dipoles with in-plane circular light (inset) reduces the relative brightness of dipoles with substantial axial components (blue curve). As a result, PSF filtration based on brightness discriminates against the contribution of axial dipoles. **(D)** Localization error for a dipole near a glass-polymer-vacuum interface, as a function of polar angle and distance from the interface (z), calculated using the selective in-plane excitation profile shown in panel (C). The refractive indices are n_1 = 1, n_2 = 1.5, and n_3 = 1.52, the pixel size is set at 213 nm, the thickness of the polymer is set to 100 nm, the thickness of the glass and vacuum layer is set to infinity, and light is collected with an air objective (0.9 NA) from the vacuum side. As shown, the dipole bias is minimal for thin films for in plane dipole (β= 90◦). The maximum effective localization error is no more than 3 nm, whereas the overall average value is less than ~ 1 nm. **(E)** Localization error for a dipole placed in a suspended vitreous ice layer with a thickness of 50 nm (the thickness of the glass and vacuum layer is set to infinity) as a function of polar angle and distance from the interface (z), calculated using the selective in-plane excitation profile shown in panel (C). The refractive indices are n_1 = 1, n_2 = 1.2, and n_3 = 1, the pixel size is set at 213 nm, and light is collected with an air objective (0.9 NA) from the vacuum side. As shown, the maximum effective localization error is no more than 1 nm, whereas the overall average value is less than ~0.3 nm. The figure was reproduced from Ref (9).

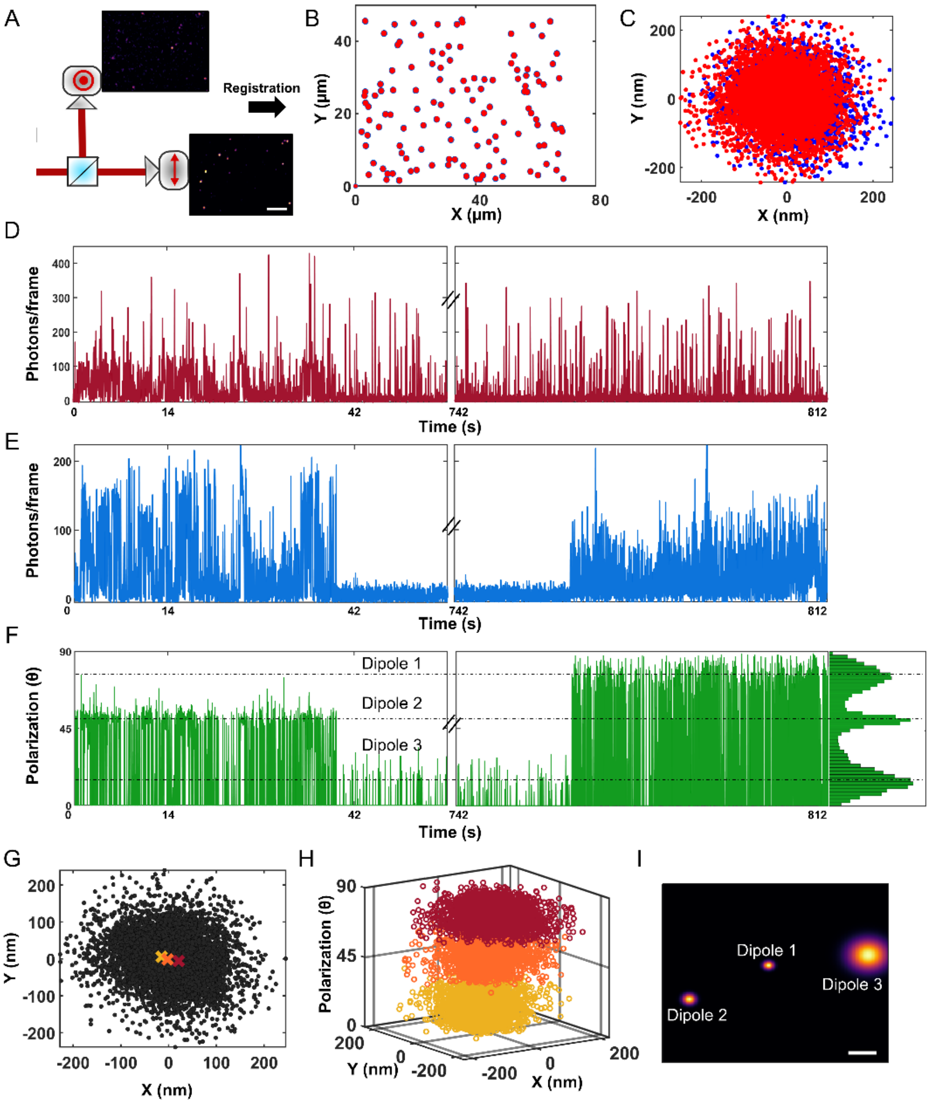

Fig. S11. Data processing. (A-C) Image registration of the two polarization channels with sub nanometer accuracy. **(A)** Raw images after polarization splitting. **(B)** Shows the registration of the entire molecules identified in one field of view. **(C)** Show the combined localizations obtained the two channels (blue and red color respectively) of one of the PSF. **(D-E)** Intensity time trace extracted from a single PSF (in panel (C)), registered on two polarization channels (red and blue). **(F)** Polarization time trace calculated from the “on events” intensities shown in (D and E). **(G)** Scatter plot of localization from all the "on" events from both polarization channels (black dots). If only the on/off time trace information is considered, the fluorophores cannot be resolved with high resolution because the signal cannot be assigned to a specific dipole, thus yield ensemble average localizations. However, by using the polarization information, the frames belonging to each dipole can be clearly identified (see panel (F), and their coordinates can then be clustered to find the center and localize each emitter with high precision (red, orange and yellow cross). **(H)** 3D plot of coordinates (x,y), and polarization, shows that in cases where the distance between the dipoles is large enough, the coordinates associated with each dipole can be revealed directly without any polarization time trace fitting. The color is coded as in G, where each color represents different dipole. **(I)** Polarization-guided, super-resolved 2D images. Using this polarization information, the frames can be clustered according to their respective dipole from the time trace, enabling generating 2D-reoslved image with angstrom precision. Scale bar is 5 nm.

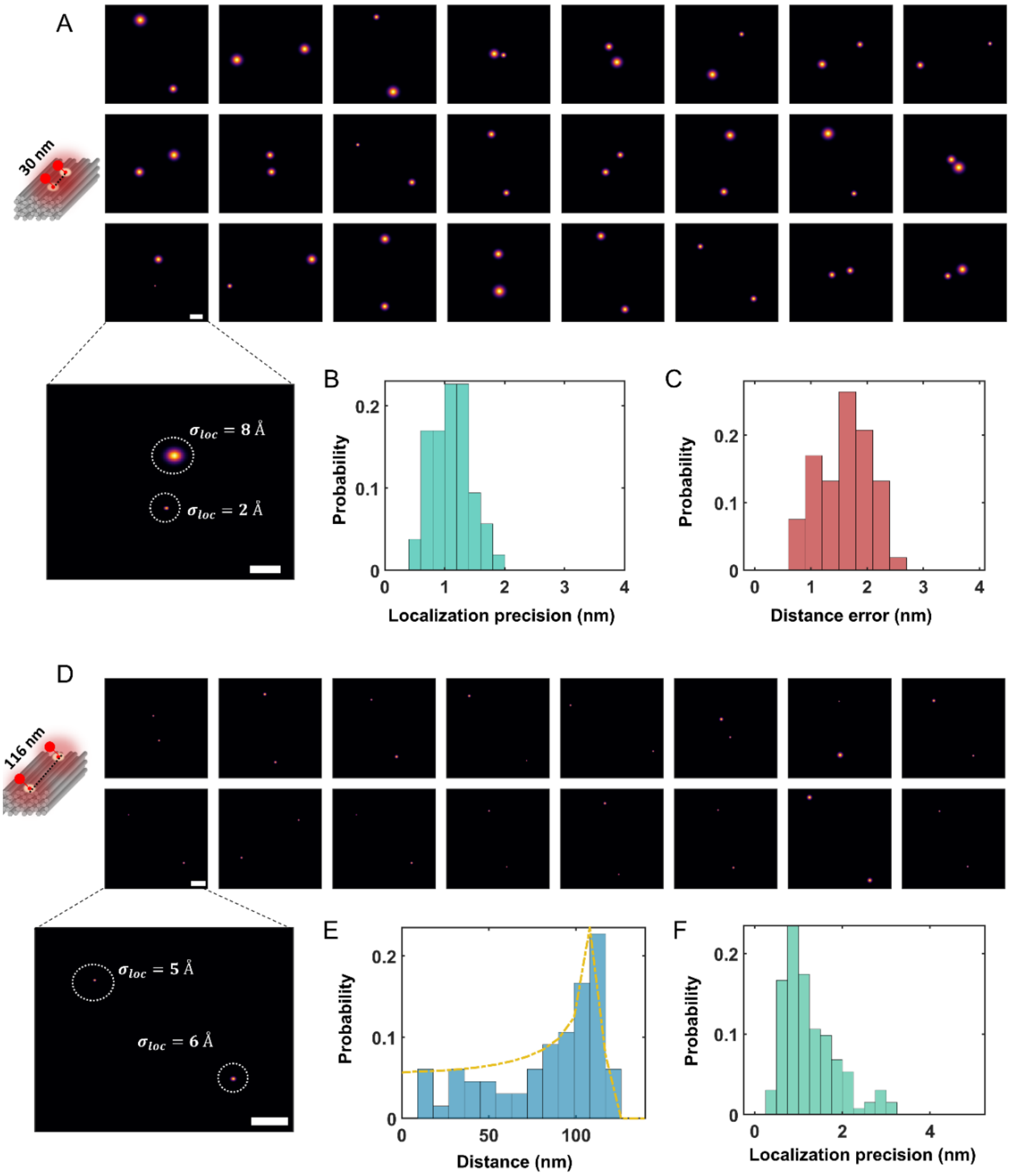

Fig. S12. DNA nanoruler analysis and validation. (A) Multiple 2D images of DNA nanoruler with two fluorophores separated by 30 nm**.** Scale bar is 5 nm. **(B)** Localization precision histogram corresponding to measurements in (A)**. (C)** Distance error estimated based on the localization precision described in Methods**. (D)** Multiple 2D images of DNA nanoruler with two fluorophores separated by 116 nm**.** Scale bar is 20 nm. **(E)** Pair-wise distance histogram (N=66) with a fit (yellow line) which takes the 3D orientations into account. The fit yields a distance of 117 nm close to the expected value of 116 nm**. (F)** Localization precision histogram for measurements in (D).

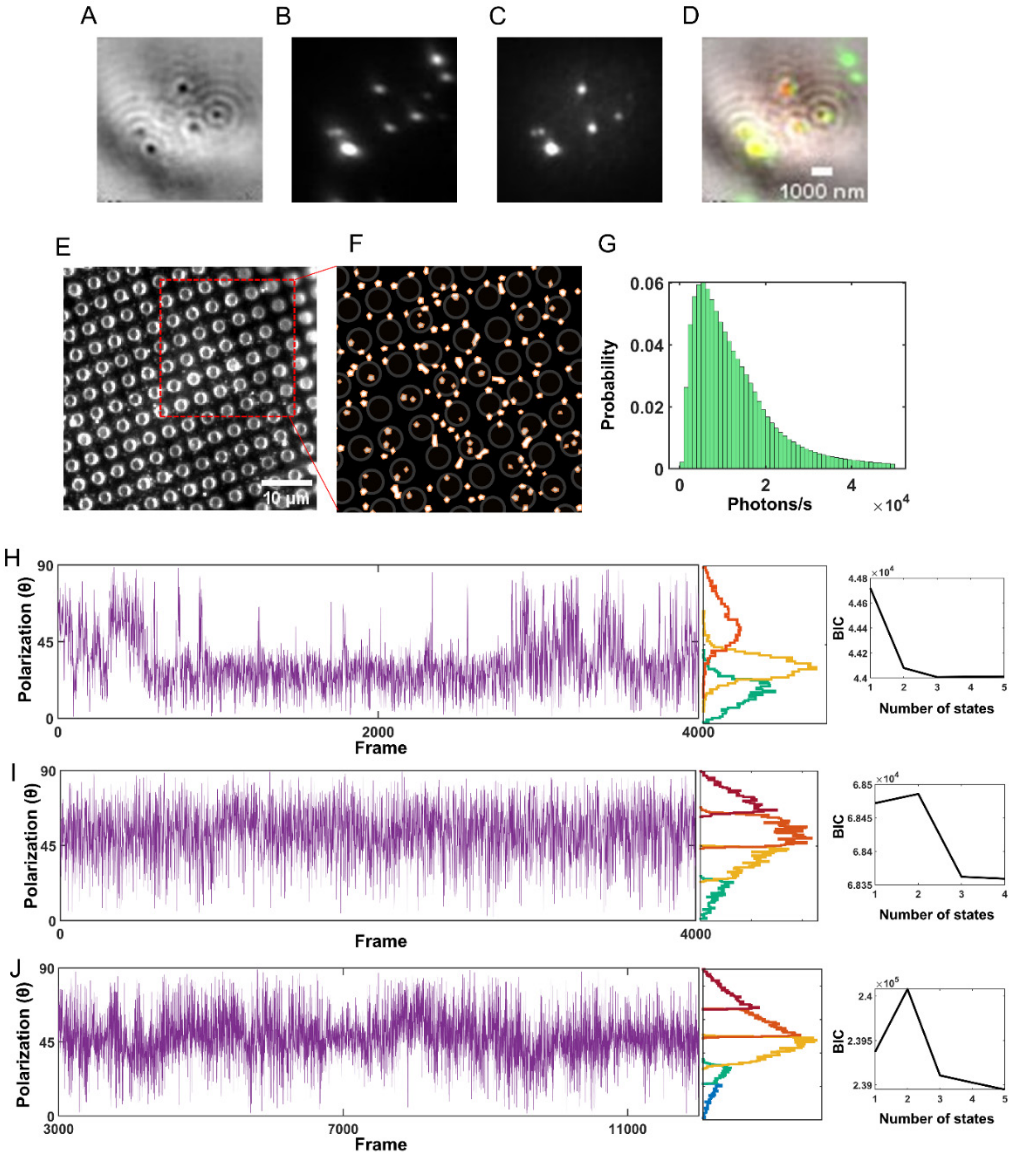

Fig. S13. αHL characterization. To validate incorporation of αHL into lipid vesicles, we tethered the prepared sample (see Methods) onto a glass substrate functionalized with poly-Lysine. The samples were then imaged in a correlative fashion using interferometric scattering (iSCAT) microscopy and fluorescence microscopy. **(A)** Signal from the iSCAT channel, clearly resolving the vesicles. **(B)** To confirm that this signal indeed comes from vesicles, we labeled them with a fluorophore (memBrite 568). Spots coincide well with those in the iSCAT channel. **(C)** Fluorescence image of αHL protein labeled with Alexa488-NHS ester. We can clearly see that the protein is localized within the vesicle signal. **(D)** Registration of all channels further confirms the colocalization of the αHL signal (red color) with the vesicle signal (green color). (**E-F)** TEM grid image of the protein in synthetic membrane recoded with our optical microscope shows sparse fluorescent molecules across the grid. (**G)** Photon distribution obtained from αHL datasets, with a median value of 10,000 photons/s. (**H-J)** Exemplary polarization time traces, showing 3-5 polarization components, as detected based on DSIC algorithm (10). We note that the signal is not continuous. It represents the on-times and discards the off-times (see Fig. S11). The histograms for each trace obtained from the segmentation analysis of the fit (see Fig. S14). The plot on the right shows the Bayesian information criterion (BIC) results, which we used to determine the optimal number of components for each time trace.

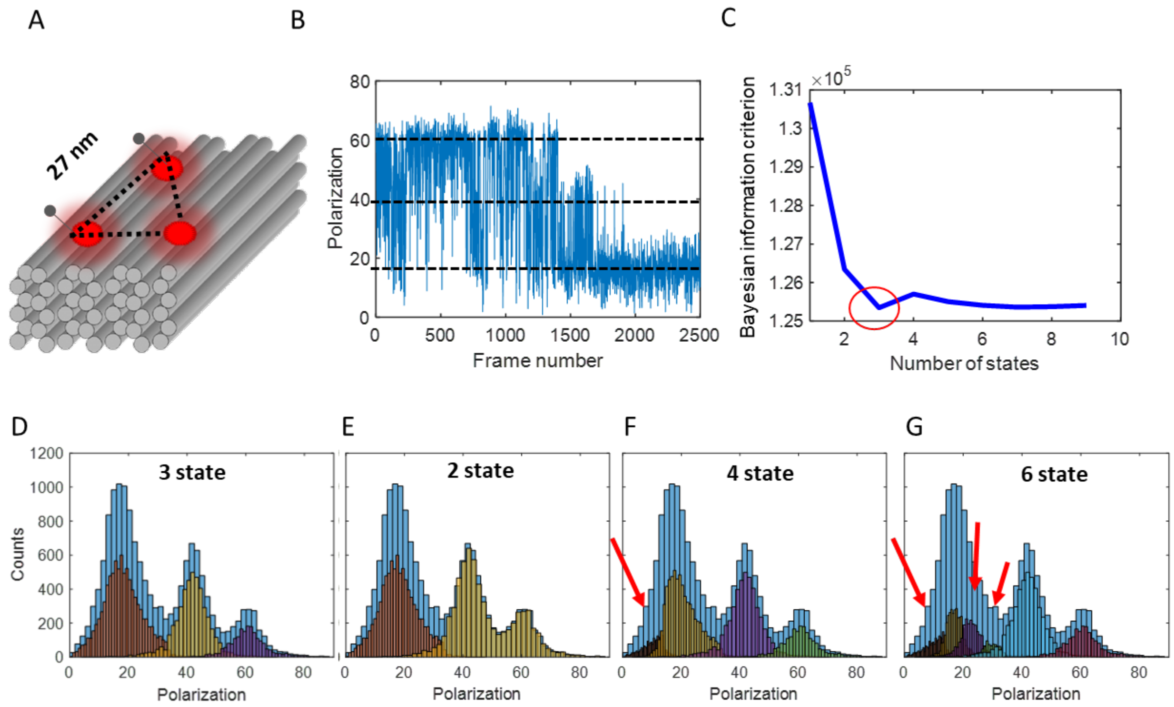

Fig. S14. Validation of data fitting using segmentation analysis. (A) Measured DNA origami structure with three fluorophores separated by 27 nm, as a benchmarking model. **(B)** Exemplary polarization time trace from the DNA molecules. The black dashed lines indicate the three resolved polarization components. **(C)** BIC of the polarization time trace in (B) indicates three components as the best model (red circle). **(D-G)** Examples of segmentation analysis for different state models. A three-state model optimally segments the data, whereas a low number of states causes the polarization components to merge. A high number, on the other hand, splits them into multiple peaks (red arrows). Clearly, the performance is improved for higher signal-to-noise ratio and less polarization uncertainty.

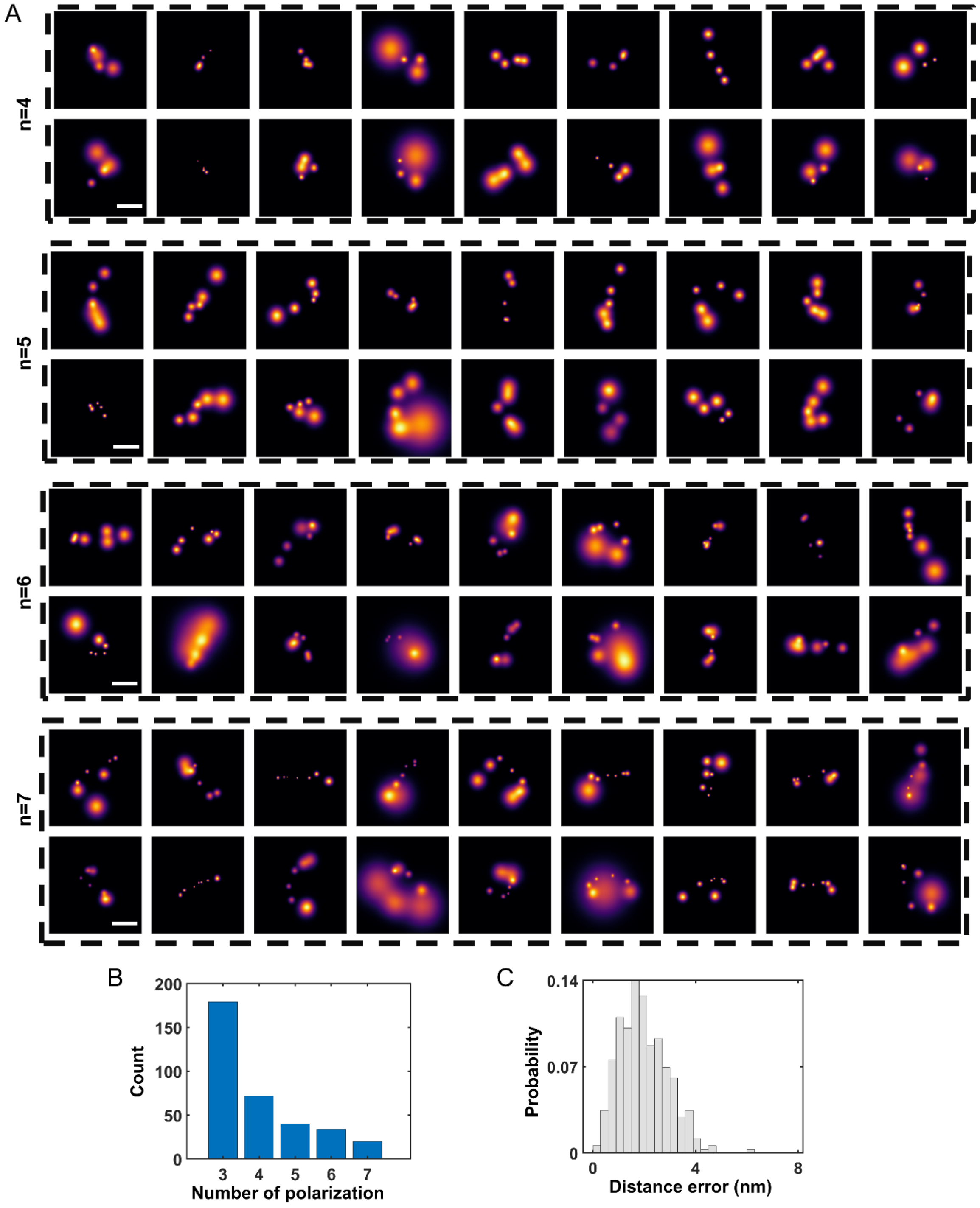

Fig. S15. 2D images obtained from an αHL sample. (**A)** Some examples of the super-resolved 2D maps obtained from the polarization trace fitted with a model based on four-seven states. Particles with the orange, green, red and purples boxes show maps with 4, 5, 6, 7 fluorophore localizations, respectively. The particles show different projections in the sample plane with some restriction (see Figure 4). The image size is 120 × 120 pixel at 0.15 nm/pixel. Scale bar is 5 nm. (**B)** Overall distribution of particles with more than two polarizations. (**C)** Distribution of distance errors from the projections in (A).

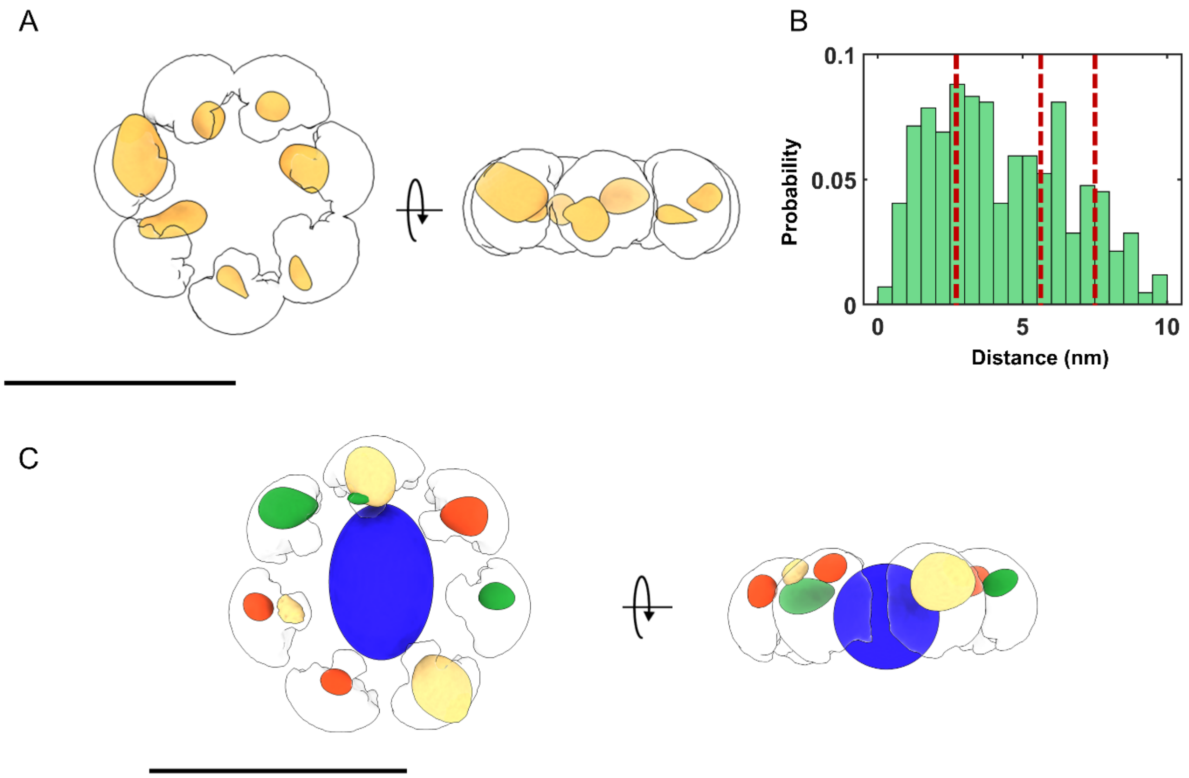

Fig. S16. 3D reconstruction of αHL sample. (A) Direct 3D reconstruction of the αHL dataset identified with seven fluorophores (*N*=20). The data indicate proper solving of the full complex, albeit a bit noisy due to low statistics (see Fig. S15). The orange spheroids are the outcome of the 3D reconstruction algorithm, which were fitted into the theoretical volume (white clouds) that is accessible to the fluorophores after taking the dye linker into account (11). We note that this is a direct calculation that requires no classification or symmetry assumptions at all. Scale bar is 10 nm. **(B)** Pair-wise distance histogram of the projections identified with seven fluorophores. The data clearly point to the correct side lengths, as expected from the αHL structural model (PDB:7ahl). **(C)** 3D reconstruction of the αHL particles identified with three fluorophores after classification into four different classes. The data show the ellipsoid structure (purple, center) used as an initial guess for the 3D reconstruction, and the outcome of the reconstruction for three of the classes. Class 2 is shown in green, class 3 in orange, and class 4 in yellow. Scale bar is 10 nm.

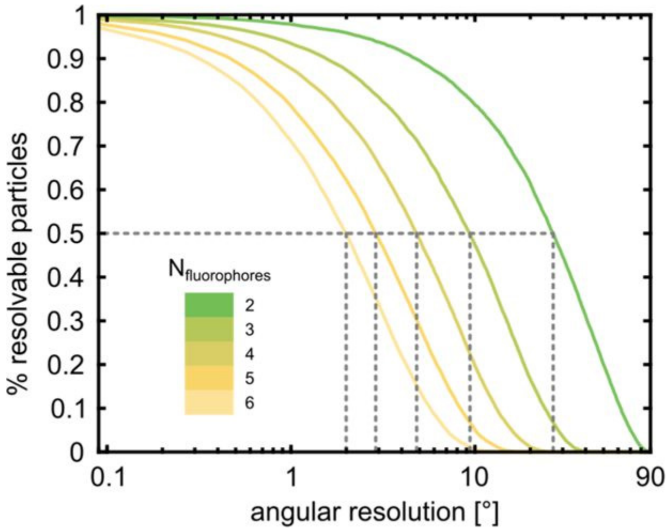

Fig. S17. Fluorophore identification using polarization-resolved imaging. The fraction of particles for which all fluorophores can be identified by the analysis of their polarization time-trace is shown as a function of the angular resolution of the fit. In order to resolve 50% of a particle with 6 fluorophores, an angular resolution of 2° is required. This requirement is relaxed with a smaller number of fluorophores. For this simulation, we assumed random dipole angles and accounted for the mapping into the [0°, 90°] interval. The figure was reproduced from ref (3) with permission; copyright 2022 eLife.

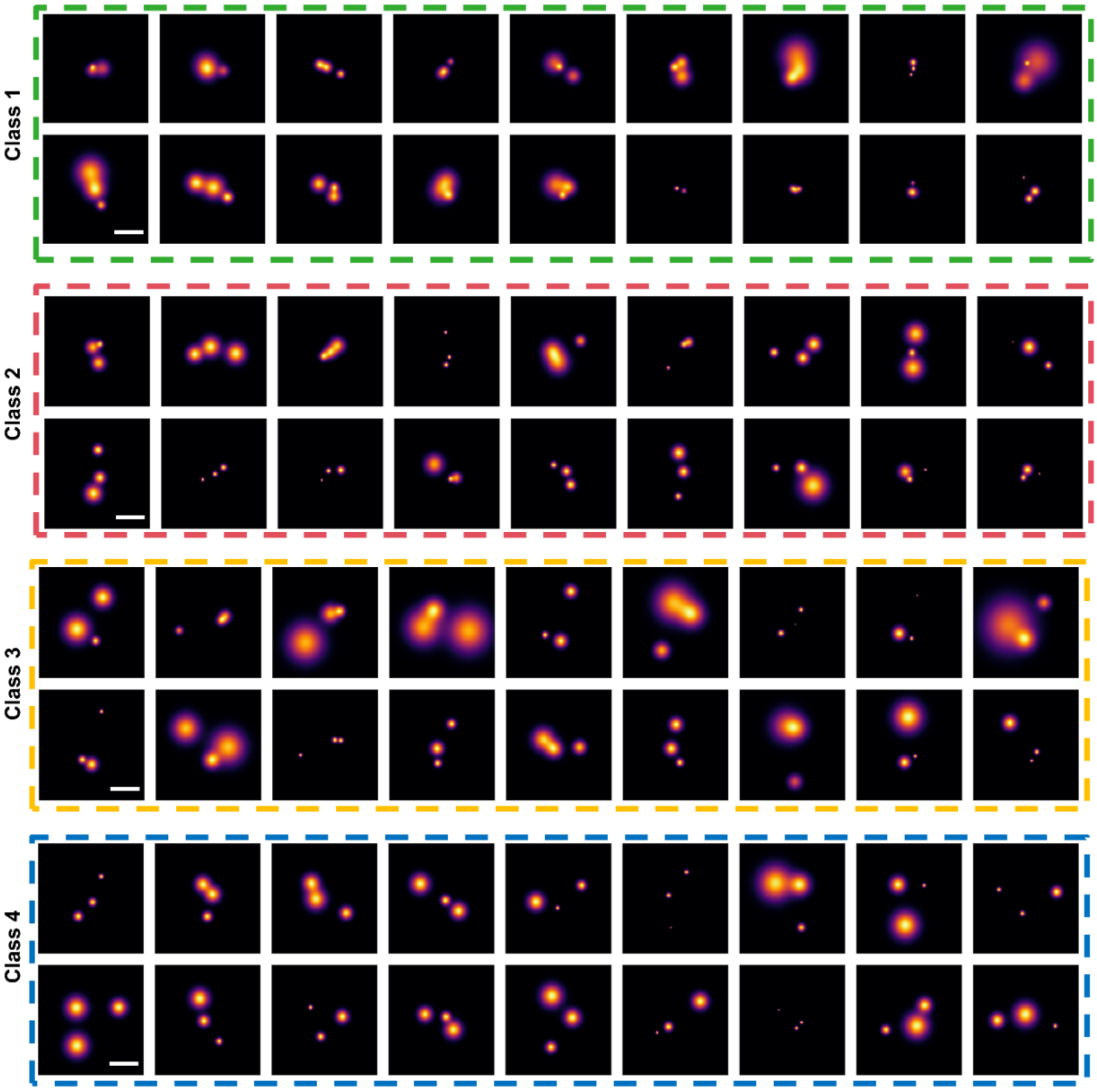

Fig. S18. 2D images of three fluorophores obtained from an αHL sample. Some examples of the 2D super-resolved maps obtained from polarization fitted with a model based on four-seven states. Particles were filtered based on localization precision below 3 nm and classified to the four inherent configurations as explained in Figure 5 of the main text (green = class 1, red = class 2, orange= class 3, blue = class 4). The image size is 120 × 120 pixel at 0.15 nm/pixel, and scale bar is 5 nm.

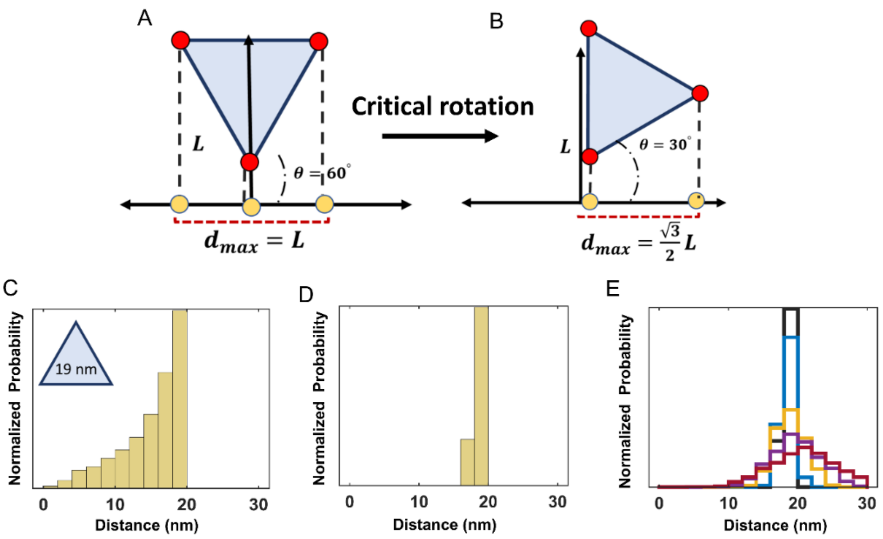

Fig. S19. Pair-wise distance histogram of simulated data for an equilateral triangle versus localization precision. In general, if we consider a single distance L between two points, one could image the absolute distance if it lies in the image plane, namely at an angle (θ) of 0°. However, in case of tilting the sample, the projected distance onto the image plane becomes skewed toward shorter values, making the distance fall within a range from [0, L]. For three points in space forming a triangle (e.g. equilateral triangle), at least one of the projected side-lengths remains close to its true value regardless of the orientation. (**A)** Scenario of an equilateral triangle with side length L. Projection onto the 2D plane yields side-length of L, and L/2. (**B)** Scenario of another special orientation. projection yields side-lengths of 0 and $\frac{\sqrt{3}}{2}L$. (**C)** Pair-wise distance histogram generated from many randomly oriented triangles with side-lengths of 19 nm. If we consider the maximum side length only from each rotated triangle, one obtains a value that is close to the true distance, resulting in a narrow distance distribution. The minimum distance in such a case is achieved at a critical angle of 30°, where the maximum distance reaches $\frac{\sqrt{3}}{2}L$. **(D)** Distance histogram after considering the maximum side-length only. The histogram is notably narrow at the right side-length. In both cases in (C-D), we assumed perfect localization precision, 0. The width of such a histogram is strongly influenced by the localization precision of the data. The lower the localization precision, the broader is the peak. **(E)** Histogram of maximum side-length as a function of localization precision. The decrease in the localization uncertainty from 0 to 0.7, 1.4, and 3 nm (color code: black, blue, orange, purple, and dark red, respectively) broadens the standard deviation of the distance distribution from 0.4 to 1.6, 3, and 5 nm, respectively. Therefore, in order to obtain valuable information on the protein scale, one must reach a precision that is superior to ~ 2 nm.

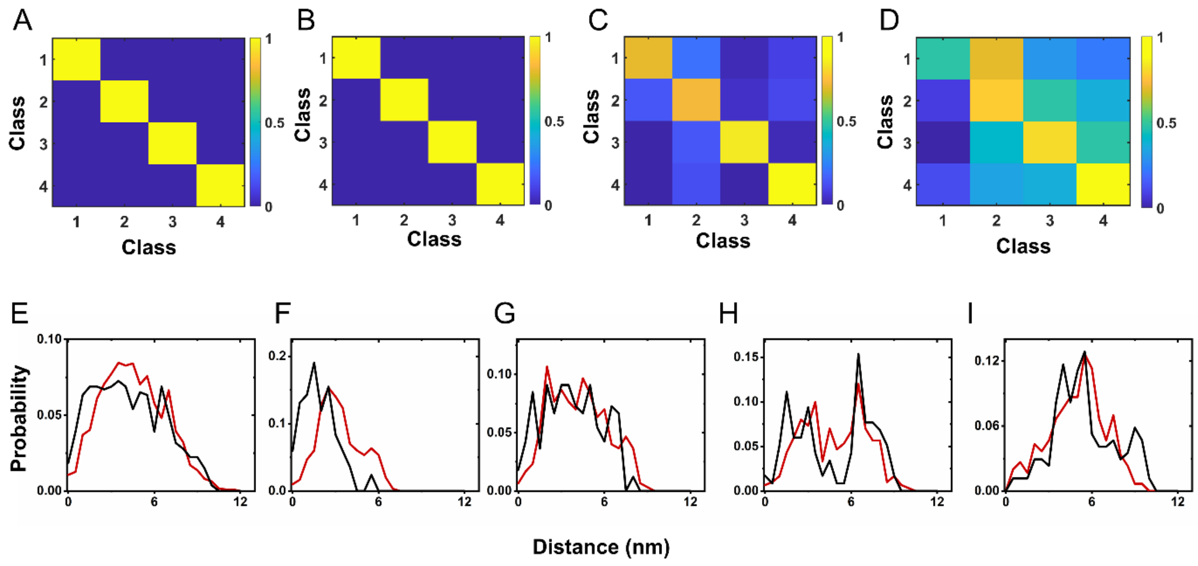

Fig. S20. Particle classification and distance histogram fit of the αHL protein. Particle classification scores for the four inherent classes of αHL (as described in Fig. 5 of the main text, and Supporting text 3). (**A)** Classification using perfect localization (0 nm precision), showing accurate classification for all classes. (**B)** Classification with 0.7 nm localization precision, maintaining accurate class assignments. (**C)** Classification with 1.8 nm localization precision, where only classes 3 and 4 maintain perfect classification. (**D)** Scenario with ±1 nm inaccuracy and 0.7 nm localization precision, showing degraded classification accuracy for classes 1 and 2. (**E-I)** Distance histograms calculated from experimental data (black curves) for particles with three fluorophores, fitted with a model accounting for particle orientation and localization uncertainty (red curves; see Methods section in main text). (E) Overall distance histogram from unclassified particles, (F) Class 1 only, (G) Class 2 only, (H) Class 3 only, (I) Class 4 only.

SI Materials and Methods

Preparation of vitrified fluorescent molecules

In this work, we tested different TEM grids. Holey support film TEM grids (R3.5/1, 200 mesh, or other holey support film shapes) were plasma cleaned using a Diener Pico 500 W plasma cleaner at 14% plasma power for 10 seconds inside a Faraday cage. Naked UltraAuFoil TEM grids were plasma cleaned at 30% plasma power for 40 seconds, while those with a 2 nm carbon coating were plasma cleaned at 24% plasma power with a 30-second incubation time. For single-molecule fluorescence measurements, 3.5 µl of 50-150 pM ATTO647N-maleimide fluorophores in a 25 mM HEPES buffer solution were loaded onto the TEM grid. The sample was then blotted after a 30-second incubation time using a Vitrobot (IV, Thermo Fisher) with standard Vitrobot blotting paper (47000-100, PLANO GmbH) and the following parameters: 1-second blotting time, -10 force, 0 wait time, 100% humidity, and 4°C. After plunge-freezing, the sample was either stored at liquid nitrogen temperature or directly transferred into our optical microscope, as described in detail in Figure 1, and Supporting text 1. Once the sample was transferred into the optical microscope, we allowed it to relax thermally and mechanically from 80 K down to 8 K for approximately 1-2 hours before starting data acquisition.

For the sample preparation procedure for the examination of vitreous ice, please see Supporting text 2.

Vitreous ice determination by cryo-electron microscopy

To examine the quality of vitrified samples (see Supporting text 2), we utilized a 300 kV Titan Krios G2 transmission electron microscope (Thermo Fisher Scientific) equipped with a K3 direct electron detector and a Bioquantum energy filter (Gatan). Initially, we created grid atlases of all samples at low magnification (34x) using SerialEM software (12). Next, the grid positions around the center and very far away from the center were imaged at eucentric height and an intermediate magnification (2250x, corresponding to approx. 3.9 nm/px) to assess local ice thickness and hole coverage. High-magnification exposures (105kx, corresponding to 0.85 Å/px) with a total dose of around 60 e/Å^2 were then recorded at the center of ice-covered holes. We analyzed the power spectra of these high-magnification exposures to detect the presence and intensity of crystalline ice diffraction rings, which are indicative of unwanted ice formation. The characteristic band between 6 and 30 Å in the power spectra can be used to evaluate the presence of crystalline ice (13, 14). Notably, some ice formation was also observed at various locations throughout the grid, regardless of the exposure state, likely due to the accumulation of transfer ice during sample preparation steps such as dewar transfer, grid clipping, and microscope transfer. For a detailed discussion on the effect of laser intensity on vitreous ice quality, please refer to Supporting text 2 and Fig. S6-S7.

Photophysics characterization

To study the photophysical behavior under different conditions (e.g., temperature and pressure), we opted to image the sample in a polyvinyl alcohol (PVA) polymer matrix. This choice was made for two reasons. First, we checked that the photophysical behavior of ATTO647N embedded in polymer was not significantly different from that in vitreous ice (see Fig. S8). Second, imaging in polymer allowed us

to measure the very same sample under all conditions, including room temperature, where ice would melt. For this purpose, we prepared a ~ 10-20 pM solution of each organic dye (ATTO647N, iFluor 647, Cy5, Alexa 647) in 50 mM HEPES buffer containing 3% PVA. The solution was spin coated onto a glass substrate and then inserted into our cryogenic optical microscope (2). To immobilize the molecules, we applied a low vacuum level for a short time (1–2 min) and then released the vacuum to reach atmospheric pressure. We first recoded the sample at RT and atmospheric pressure. The chamber was then evacuated to ~ 10^{-4} mbar, and a different field of view (FOV) of the same sample was recoded. Next, we cooled down the chamber using LHe in combination with a heater on the cold finger, which is controlled via a dedicated temperature controller (Lakeshore) in order to balance the temperature at 77 K. After temperature stabilization (~ 1 h), we recoded another FOV of the same sample. Finally, we turned off the heater and allowed the system to cool further to LHe temperature ~ 8 K and to relax before acquiring data. In all conditions, the sample was illuminated with a laser intensity of ~ 0.65 kW/cm^2 and recorded at a frame rate of 70 Hz. The recorded data were then analyzed using our custom-written code for detecting and clustering the molecules (2). The off-on ratio from each PSF/molecule was calculated by dividing the full intensity trajectory (until bleaching point) into 50-frame bin size. For each bin, we calculated the ratio of off-frames to on-frames, then averaged these values across all bins. The final off-on ratio was plotted using a violin plot or as histogram. We note that our imaging frame rate of 70Hz for a FOV of 48 µm x 71 µm limits the temporal resolution of on-times to 14 ms.

GUV preparation

GUVs were prepared following previously published protocol (15). To prepare fluorescently labeled GUV's, we dissolved 5mg 1,2-Dimyristoyl-sn-glycero-3-phosphoethanolamin – ATTO647N (DMPE-ATTO647N, AD 647N-191, ATTO-TEC) into 500 µl of 20:80 methanol chloroform mixture to obtain stock solutions of 10mg/ml and 0.1 mg/ml. We aliquoted the solution to ~100 µl volume, and then desiccated them over night at 1e-2 mbar and RT. Then we sealed the tubes and stored them at -20 °C. To prepare the GUV lipid mix, we took 35 µl of 0.1 mg/ml DMPE ATTO-647N and we mixed it with 200 µl matrix lipids 1,2-dioleoyl-sn-glycero-3-phosphocholine (DOPC, 850375 Avanti Polar Lipids) (10mg/ml) and 1765 µl pure chloroform to arrive at a final lipid concentration of 1 mg/ml. DOPC were prepared in a similar way but by mixing the powder with pure chloroform. We then prepared 25 mm coverslips which were pre-washed with 100% ethanol and then plasma cleaned at 100% plasma power for 10 min. We applied 150 µl of 5% poly-vinyl-alcohol (PVA) (8.14894, Sigma Aldrich) and spin coated at 1200 rpm for 30 s. We then dried the sample at 70 °C for ~ 1.5 h. We applied 5 µl GUV lipid mix and spread the solution across the surface. We then dried the sample via desiccation at 10^{-2} mbar for 15-30 min. Next, we added a chamber ring and glued it onto the coverslip with dentist glue. Finally, we added 300 µl aqueous buffer solution and incubated for ~ 2 h. GUV formation was monitored under standard fluorescence microscope. To image the vitrified GUV's using our optical microscope, a holey support film TEM grid was incubated with 5% PVA and then blotted manually and the solution was allowed to dry. We then applied 3.5 µl of the GUV solution and let it spread on the grid for 30 s, followed by blotting with the following parameters: 3s blotting time, -5 force, 0 wait time at 100% humidity and 4 °C. Samples

were then transferred into our optical microscope as described before. We recorded several images of the grid which showed intact GUV's, using 645 nm laser at ~ 0.2 kW/cm^2 laser intensity and 32 Hz frame rate.

SUV preparation and SLB formation

Small unilamellar vesicles (SUVs) were prepared following a previously published protocol (16). To generate fluorescently labeled SUVs, we mixed 35 or 3.5 µl DMPE-ATTO647N (0.1 mg/ml) with 200 µl DOPC (10 mg/ml) for different fluorophore densities. We dried the solution onto the inner surface of the vial followed by desiccation overnight. We added 2 ml aqueous buffer and vortexed to generate a milky suspension. To obtain homogenous SUVs with well-defined diameter, we extruded the milky suspension sample through a 200 nm pore filter (Whatman Anotop) 37 times. In the case of αHL protein, we produced SUVs from 1,2-diphytanoyl-sn-glycero-3-phosphocholine (850356 Avanti Polar Lipids) following the same procedure. TEM grids with 2nm carbon on top were incubated with SUV solution for 5 min, followed by quick dip in a clean buffer. We applied 3.5 µl clean buffer, blot, and plunge-froze as indicated previously. We used a Teflon sheet facing the side of the membrane and standard blotting paper on the other side. The sample was then transferred and imaged as mentioned previously.

smURFP purification and imaging

The gene expression smURFP obtained as a gift from Erik Rodriguez & Roger Tsien (Addgene plasmid # 80341 ; http://n2t.net/addgene:80341 ; RRID:Addgene_80341) (17). One Shot TOP 10 competent cells (C404010 Thermo Fisher) were transformed with protein vectors and were grown overnight in 10 ml lysogeny broth (LB) + ampicillin. The 10 ml were added to warm 100 ml LB in a 250 ml beaker for large-scale purification. Expression was induced after 2 h incubation by adding 0.2% arabinose, and the cells were incubated at 37 °C overnight at 225 rpm shaking. Following expression, bacteria were harvested and the proteins were purified on a Ni-NTA resin (GE Healthcare) with an elution step involving 250 mM imidazole. This purification was followed by buffer exchange using Amicon® Ultra Centrifugal Filter, 50 kDa to remove imidazole from the solution. The sample purity and protein fluorescence were assessed by native-page and SDS-page gel. The protein was aliquoted and stored at -80 °C. For imaging, UF-2C TME grid were plasma cleaned as mentioned previously. 150 pM of smURFP was applied on a UF-2C TME grid and blotted at -2 force. The sample was then transferred and imaged with laser intensity at 0.3 kW/cm^2 and 70Hz frame rate.

DNA nanoruler imaging

30 nm DNA nanoruler was purchased from Tilbit nanosytems GmbH, and 116 nm was purchased from GATTAquant GmbH. In both cases, ATTO647N was used as label. For imaging, UF-2C TME grid were plasma cleaned at 15% power for 25 s in a Faraday cage. DNA sample was diluted by a factor of 4 from the stock sample as received from the manufacture, in a buffer contacting 20 mM $MgCl_2$. 3.5 µl of the sample was applied onto the TEM grid and blotted at -5 force for 2 s. The sample was transferred and imaged with laser intensity at 0.65 kW/cm^2 and 50-70Hz frame rate.

Alpha-hemolysin labeling and lipid reconstitution

C-terminal His-tagged αHL protein from *Staphylococcus aureus* was purchased from Hölzel Diagnostika Handels GmbH (catalogue number CSB- YP639324FLFc7- 200). The protein was dissolved in 25 mM HEPES buffer, 25 mM KCl at pH 8 to a concentration of 10 μM. The sample was next desalted using size-exclusion column PD SpinTrap G-25 (Cytiva, 28918004) to remove older buffer residuals. The protein was specifically labeled via the histidine linker on the C-terminal side of the protein with the dye HIS Lite™ iFluor™ 647 Tris NTA-Ni Complex which was purchased from AAT Bioquest (catalogue number 12618). The protein was reacted with dyes at a ratio of 1:2 respectively for 2 h at RT. The protein was then desalted from the excess of dyes using the same desalting column. The labeling efficiency was estimated using an absorption spectrometer (Nanodrop 2000, ThermoFischer), confirming ~ 100% labeling efficiency. However, as the sample is diluted and washed further during the sample preparation, the labeling efficiency is expected to decrease since the labeling is not covalent. The sample was aliquoted, flash frozen and stored at -80 °C. To generate the protein lipid construct, we followed previously published protocols (18). First, we incubated the protein with a pre-made 200 nm SUVs at 38 °C for 1-2 hr. Then we performed size exclusion to purify the SUVs only and remove unbound free protein. We validate the incorporation of αHL into vesicles via correlative interferometric scattering (iSCAT) and florescence imaging (see Fig. S13). We incubated plasma-cleaned UF TEM grid with 2 nm carbon on top with the protein-lipid mixture for 5 min at RT. The sample was then dipped several times in clean buffer before plunge-freezing. In the plunge-freezing process the sample was blotted with a teflon paper on the side facing the lipids and standard paper on the other side. We note that some residual SUVs might still be present on the sample as the washing was very gentle. In addition, as the concentration of αHL is lower than that at the labeling stage, some of the fluorophores might dissociate as the binding of the fluorophore to the 6x Histidine tag is in the nM range.

Optical setup

Our experiments were conducted in a custom-built cryogenic microscope, which utilizes a Janis ST-500 flow cryostat to maintain liquid helium temperatures. To facilitate the insertion and removal of vitrified samples, we modified the microscope's cold finger to accommodate a dovetail-shaped sample cartridge, allowing for high-vacuum and low-temperature operation (see details in Figs. S1-4). The mechanical drift of the microscope was measured to be approximately 5 nm/min on average. Additionally, the temperature at the cold finger was verified to be stable at 8.23 K, regardless of the laser intensity used. The temperature characterization of our sample cartridge is presented in Figs. S2-3 and described in detail in the supporting information. The optical setup is identical to that used in our previous work, as described in Refs. (2, 3). In brief, samples are loaded onto the cold finger and imaged using a 0.90 NA objective lens and 2 mm focal length (MPLAN 100x, Mitutoyo) mounted in vacuum, which focuses the fluorescence onto two separate EMCCD cameras (Andor iXon) in a polarization-resolved configuration. An imaging lens of 150 mm focal length was used to achieve a nominal effective pixel size of 214 nm. The experimental pixel size was determined to be 227 nm, using a microfabricated reference structure. To achieve fast acquisition rates, the field of view was set to 211 x 313 pixels. The laser intensity was set to approximately 0.65 kW/cm^2, and images were recorded with an exposure time of 14 ms, which is more than five times faster than the typical off-time. This allows us to capture

individual fluorescence bursts and minimize the likelihood of overlapping emission from multiple fluorophores in a single frame. For each field of view, we collected a total of 50,000 to 100,000 frames, depending on the specific experiment

Image analysis

We employed custom-written MATLAB software, as described in our previous works (2, 3), to analyze the raw image stacks from two polarization channels. However, instead of median filtering, we used the band-pass filter algorithm (59) to detect the PSFs, with a low pass value of 0.3 and a high pass value of 3. In the case of αHL, a large background was observed around the holes (see Fig. S13). As a result, we selected particles that were distinct and sufficiently far from the holes. To further investigate the polarization time-traces of multi-fluorophore particles, we utilized a routine based on the DISC algorithm (10).This algorithm is fully unsupervised and requires no training data. It employs change point analysis and a k-mean clustering approach to determine the number of components in the polarization time trace (see previous implementation, Ref. (3). The algorithm employs a statistical measure to determine the optimal number of components using Bayesian information criterion (BIC).

We validated the modeling using segmentation analysis, which we performed visually for each dataset. Here, the best-fit model is used to generate sub-histograms by assigning each point in the time trace to its corresponding polarization component. Correct modeling yields distinct peaks in the segmented polarization histogram, whereas incorrect modeling merges components due to misassignment (see Fig. S14). In this case, the segmented peaks must also fulfill physical constraints. One important feature is that the width of each component should be close to the shot noise limit below ~5° (see Fig. 3J).

We note that there is ambiguity in the polarization time trace analysis, in particular for traces with low SNR. In the worst-case scenarios of poor modeling, the localization of multiple fluorophores in space would be merged, thereby reducing the overall localization precision. Such data are, however, sharply filtered out in our analysis pipeline as we only consider particles with an average precision below 2 nm. High photon collection schemes would improve the SNR and thus reduce the usage of such computational approaches, as the polarization peaks would be easily detected without the need for sophisticated processing (see Fig. S11, and Fig. S14 for example). Deep learning approaches trained on ground truth datasets, such as those based on DNA origami, could yield a more accurate representation of the data.

Nevertheless, the approach enables us to determine the number of polarization states per PSF, which corresponds to the number of fluorophores per PSF or protein particle. The underlying principle is that the dipole orientation of each fluorophore at 8 K is random but fixed (3), allowing us to annotate each fluorophore over time and localize it with high precision beyond the diffraction limit through coordinate clustering (3). By assigning a 2D Gaussian function to each localized polarization/fluorophore, with a width determined by the respective localization precision, we reconstructed 2D super-resolved images. These images provide different projections of the protein molecules within the sample. The 2D images are then subjected to further analysis, including distance measurements, classification, and 3D reconstruction, as outlined below.

Single-molecule 2D image analysis of αHL

To classify the experimental 2D maps of the αHL, we followed a similar approach as reported in Ref. (3). Here particles with three fluorophores inherently generate 4 different classes (see Figure 5C). We simulated multiple 2D projections of the protein spanning all possible 3D orientations using the theoretical structural model (PDB: 7ahl) for each class separately. We then performed 2D cross-correlation of the experimental and simulated maps of all classes. The correlation score, which ranges from 0 to 1 with the latter indicating a 100% match, was used to assign a class to each experimental image. In this case, an image is assigned to a certain class if the correlation score of this class is the highest score among the others. See Fig. S18-20 for additional analysis.

Distance error estimation

The error on the measured distances were calculated as described previously (19) in an error propagation manner following $\sqrt{\sigma_1^2 + \sigma_2^2}$, where $\sigma_{1,2}$ indicate the localization precision of positions 1 and 2, respectively. The latter quantities are calculated as $\sigma_{1,2} = \sqrt{\frac{\sigma_x}{\sqrt{N}} * \frac{\sigma_y}{\sqrt{N}}}$, where $\sigma_{x,y}$ is the standard deviation of the position in the x and y directions, respectively, and *N* is the number of localizations per position.

Distance fitting

For more quantitative analysis of the pairwise distances, we used a model described in (2, 20). In short, we convolve the Rician distribution of the distance between two spots with finite localization uncertainty, considering the projection onto the image plane to be a cosine function.

Estimation of particle orientation

To determine the orientation of our particles in 2D space, we used a pipeline similar to that described in our previous work (19). Here, however, we employed triangular model for each class separately, as depicted in Fig. 5C.

Optical tomography and 3D reconstruction

We selected particles that exhibited a high degree of similarity to the simulated 2D projections, as evidenced by a cross-correlation score greater than 0.8 and a localization precision better than 2 nm. The 2D maps of the particles were then normalized and the median of the full width at half-maxima was taken as the localization uncertainty for all particles, in order to obtain a spherical volume. The resulting 2D maps of the αHL protein were constructed on a 120x120 grid with a pixel size of 1.5 Å. The 2D projections were then used as input for the 3D reconstruction subspaceEM algorithm (21), with an elliptical Gaussian serving as an unbiased initial structural model. We ran the algorithm for 100 iterations using default settings. The 3D volumes were then refined by fitting them to crystal structures and maps of the dye location (11), and edited using ChimeraX (22). To assess the resolution of the 3D reconstructions, we computed the Fourier shell correlation (FSC) curves using the FSC server provided by the Protein Data Bank in Europe (https://www.ebi.ac.uk/pdbe/emdb/validation/fsc/). This involved

dividing the 2D image data into two equal sets, reconstructing the 3D volumes independently using the previously determined 3D volume as an initial model, and then aligning the two reconstituted volumes to calculate the FSC. The resolution was determined based on the half-bit criterion (23), which provides a reliable estimate of the achievable resolution.

Data visualization and analysis

All data analysis done using MATALB Mathworks software. Some of the figures were plotted using OriginPro 2020. Protein structures and 3D reconstitution volumes were processed using PyMOL v2.4 and ChimeraX v1.7.1. Cryo-EM images were visualized using IMOD.

Supporting protocol

Procedure at a Glance

Forward direction: Preparation chamber to optical microscope.

Step	Description
1	Evacuate the cryogenic optical microscope to a primary vacuum <10^{-5} mbar. Start the flow of liquid helium into the cryostat until reaching 4 K. The vacuum at this stage goes down further to <10^{-6} mbar. At this point, the optical microscope is ready for sample transfer. Keep the vacuum pump on and stabilize the helium flow to maintain a constant temperature.
2	Prepare the plunge-frozen TEM grids. Put them into storage in liquid nitrogen in a sealed grid box.
3	Purge the preparation chamber and the high-vacuum shuttle with dry nitrogen for ~10-20 minutes to minimize moisture.
4	Connect the transfer shuttle to the preparation chamber with its gate valve closed. Evacuate the transfer shuttle to a primary vacuum <10^{-5} mbar and fill the dewar with liquid nitrogen. Wait for the temperature to stabilize at 90 K. From this point on, keep filling the dewar along all the procedure until the transfer is finished. (15 min)
5	Prepare the preparation chamber. Move the glass vessel to the upper position and insert a cartridge into the cold stage adapter. Fill the vessel with liquid nitrogen until the cartridge is completely immersed and boiling stops. (5- 10 min)
6	Quickly move the grid box from the storage container to the vessel. Insert a tweezer and a screw driver and wait for their tips to be cold. Maintain a constant flow of dry nitrogen gas through the chamber. (~ 1 min)
7	Carefully remove a grid from the grid box with the tweezer and place onto the recess in the cartridge. Fix the grid with the pre-cooled clamp by tightening the screw. (1- 5 min)
8	Close the gate valve of the transfer shuttle and break the vacuum near the gate valve of the preparation chamber. Allow dry nitrogen to fill the atmosphere and the pipe of the vacuum pump to reduce the moisture. (3 - 5 min)
9	Place the anti-contaminator plate on top the sample cartridge. Close the top part of the sample loading chamber and lower the glass vessel. Stop the dry nitrogen flow and start evacuating to a primary vacuum of <10^2 – 10^0 mbar (depend on the level of LN in the vessel), open the gate valve of the shuttle and retrieve the cartridge with the manipulator. (5 min). There is a risk of water vapor condensation on top of the cartridge at this stage. While such condensation does not affect the quality of optical measurements, it should be avoided in correlative LM/EM studies. To achieve that, future work will implement a mechanical shutter on the sample cartridge.
10	Close the gate valve of the preparation chamber and switch on the turbo-molecular pump. Evacuate to a secondary vacuum of <10^{-6} mbar. (3 - 4 min)
11	Wait for the temperature and pressure to stabilize, then close the gate valve and switch off the pump. The vacuum in the shuttle is now maintained by the cold stage.
12	Connect the shuttle to the interface on the microscope (KF50 flange) and switch on the pump. Wait for the pressure in the differential pumping stage to reach <10^{-5} mbar before opening the gate valves. (~ 2 min).
13	Open the gate valve of the shuttle first and let the pressure stabilize. Then open the gate valve of the microscope. (~ 2 min)
14	Insert the cartridge into the microscope cold stage adapter with the linear manipulator. Disconnect by rotating the manipulator to remove the screw from the thread in the cartridge. (~ 1 min)
15	Retract the manipulator, close the gate valves, vent the differential pumping stage and disconnect the shuttle from the microscope. Also disconnect the vacuum pump from the optical microscope.
16	Wait for at least 1-2 h before starting image acquisition.

Reverse direction: Optical microscope to preparation chamber.

Step	Description
1	Connect the transfer shuttle to the optical microscope. Evacuate the transfer shuttle to a primary vacuum $<10^{-5}$ mbar and fill the dewar with liquid nitrogen. Wait for the temperature to stabilize at 90 K. From this point on, keep filling the dewar along all the procedure until the transfer is finished. (~ 15 min, if the transfer shuttle was warm)
2	Open the gate valve of the shuttle first and let the pressure stabilize. Then open the gate valve of the microscope. (~ 2 min)
3	Load the sample cartridge and park it on the cold finger of the transfer shuttle using the linear manipulator and then close the gate valves.
4	Vent the differential pumping stage and disconnect the shuttle from the microscope.
5	Connect the transfer shuttle to the preparation chamber.
6	Cool down the working platform inside the preparation chamber with LN. Keep purging N_2 gas in the preparation chamber to reduce moisture.
7	For high-vacuum transfer, exchange the liquid nitrogen vessel with an empty one and immediately apply vacuum to reach 10^{-4} mbar. The working platform temperature will remain cold, below 130 K. Alternatively, to transfer the sample back more quickly, the process can be performed at a higher pressure by keeping some LN in the vessel. This method is suitable if a shutter is installed on the sample cartridge to prevent water vapor condensation.
8	Open the gate valves and transfer the cartridge to the cold finger inside the preparation chamber.
9	Close the gate valve of the transfer shuttle and immediately break the vacuum with some N_2 gas. Immediately pore fresh LN into the vessel to cover the sample cartridge.
10	Unclamp the grid and store it in TEM grid box for subsequent Cryo-EM measurement.

Legends for Movies S1 to S8

Movie S1: Step-by-step transfer of vitrified samples at high vacuum and cryogenic temperature. The video shows the entire sample transfer process. The video has been sped up 2 times to reduce its size.

Movie S2: Breaking of the Carbon Mesh on the TEM Grid. The video shows the carbon film tearing apart after laser illumination at an intensity of ~ 1 kW/cm^2.

Movie S3: Fluorescence imaging of ATTO647N in vitrified aqueous solution on carbon mesh TEM Grid. The video was acquired at 70 Hz using one camera after polarization splitting.

Movie S4: Fluorescence imaging of ATTO647N in vitrified aqueous solution on UltrAuFoil TEM grid. The video was recorded on one of the cameras after polarization splitting at 70 Hz.

Movie S5: Fluorescence imaging of smURFP in vitrified aqueous solution on UltrAuFoil TEM grid. The data was recorded on one of the cameras after polarization splitting at 70 Hz.

Movie S6: Fluorescence imaging of DNA nanoruler (30 nm, tilibit nanosystems GmbH) in vitrified aqueous solution on UltrAuFoil TEM grid. The data was recorded on one of the cameras after polarization splitting at 70 Hz.

Movie S7: Fluorescence imaging of αHL sample reconstituted in SUV on UltrAuFoil TEM grid. Fluorescence images of vitrified αHL reconstituted in SUVs after incubation on UltrAuFoil TEM grid with 2 nm carbon on top. The video shows sparse point spread functions with good on-off ratio. Data were recorded at 70 Hz using one camera after polarization splitting.

Movie S8: 3D reconstruction of αHL sample. The video shows the final 3D reconstruction of each αHL class labelled with three fluorescent molecules. The 3D reconstructions were then merged to generate the full heptameric assembly. Scale bar is 10 nm.

SI References

1. S. Tacke *et al.*, A Versatile High-Vacuum Cryo-transfer System for Cryo-microscopy and Analytics. *Biophys J* **110**, 758-765 (2016).
2. D. Böning, F.-F. Wieser, V. Sandoghdar, Polarization-Encoded Colocalization Microscopy at Cryogenic Temperatures. *ACS Photonics* **8**, 194-201 (2021).
3. H. Mazal, F. F. Wieser, V. Sandoghdar, Deciphering a hexameric protein complex with Angstrom optical resolution. *Elife* **11**, e76308 (2022).
4. P. D. Dahlberg, D. Perez, C. W. Hecksel, W. Chiu, W. E. Moerner, Metallic support films reduce optical heating in cryogenic correlative light and electron tomography. *Journal of Structural Biology* **214**, 107901 (2022).
5. M. W. Tuijtel, A. J. Koster, S. Jakobs, F. G. A. Faas, T. H. Sharp, Correlative cryo super-resolution light and electron microscopy on mammalian cells using fluorescent proteins. *Sci Rep* **9**, 1369 (2019).
6. C. J. Russo, L. A. Passmore, Ultrastable gold substrates for electron cryomicroscopy. *Science* **346**, 1377-1380 (2014).
7. S. Mojiri *et al.*, Effects of base temperature, immersion medium, and EM grid material on devitrification thresholds in cryogenic optical super-resolution microscopy. *J Struct Biol* **217**, 108231 (2025).
8. J.-P. Wieferig, D. J. Mills, W. Kühlbrandt, Devitrification reduces beam-induced movement in cryo-EM. *IUCrJ* **8**, 186-194 (2021).
9. F.-F. Wieser (2023) Single-particle cryogenic light microscopy. (Dissertation, Friedrich-Alexander-Universität Erlangen-Nürnberg, Erlangen).
10. D. S. White, M. P. Goldschen-Ohm, R. H. Goldsmith, B. Chanda, Top-down machine learning approach for high-throughput single-molecule analysis. *eLife* **9**, e53357 (2020).
11. S. Kalinin *et al.*, A toolkit and benchmark study for FRET-restrained high-precision structural modeling. *Nature Methods* **9**, 1218-1225 (2012).
12. D. N. Mastronarde, Automated electron microscope tomography using robust prediction of specimen movements. *Journal of Structural Biology* **152**, 36-51 (2005).
13. N. Biyani *et al.*, Focus: The interface between data collection and data processing in cryo-EM. *Journal of Structural Biology* **198**, 124-133 (2017).
14. G. McMullan, K. R. Vinothkumar, R. Henderson, Thon rings from amorphous ice and implications of beam-induced Brownian motion in single particle electron cryo-microscopy. *Ultramicroscopy* **158**, 26-32 (2015).
15. H. Stein, S. Spindler, N. Bonakdar, C. Wang, V. Sandoghdar, Production of Isolated Giant Unilamellar Vesicles under High Salt Concentrations. *Frontiers in Physiology* **8** (2017).
16. C.-L. Hsieh, S. Spindler, J. Ehrig, V. Sandoghdar, Tracking Single Particles on Supported Lipid Membranes: Multimobility Diffusion and Nanoscopic Confinement. *The Journal of Physical Chemistry B* **118**, 1545-1554 (2014).
17. E. A. Rodriguez *et al.*, A far-red fluorescent protein evolved from a cyanobacterial phycobiliprotein. *Nature Methods* **13**, 763-769 (2016).
18. P. B. Stranges *et al.*, Design and characterization of a nanopore-coupled polymerase for single-molecule DNA sequencing by synthesis on an electrode array. *Proceedings of the National Academy of Sciences* **113**, E6749-E6756 (2016).
19. H. Mazal, F. F. Wieser, D. Bollschweiler, A. Schambony, V. Sandoghdar, Cryo-light microscopy with angstrom precision deciphers structural conformations of PIEZO1 in its native state. *Sci Adv* **11**, eadw4402 (2025).
20. S. Niekamp *et al.*, Nanometer-accuracy distance measurements between fluorophores at the single-molecule level. *Proceedings of the National Academy of Sciences* **116**, 4275-4284 (2019).
21. N. C. Dvornek, F. J. Sigworth, H. D. Tagare, SubspaceEM: A fast maximum-a-posteriori algorithm for cryo-EM single particle reconstruction. *Journal of Structural Biology* **190**, 200-214 (2015).

22. T. D. Goddard *et al.*, UCSF ChimeraX: Meeting modern challenges in visualization and analysis. *Protein Science* **27**, 14-25 (2018).
23. M. Van Heel, M. Schatz, Fourier shell correlation threshold criteria. *Journal of Structural Biology* **151**, 250-262 (2005).
